# The Presence of a Cyclohexyldiamine Moiety Confers Cytotoxicity to Pentacyclic Triterpenoids

**DOI:** 10.3390/molecules26072102

**Published:** 2021-04-06

**Authors:** Sophie Hoenke, Martin A. Christoph, Sander Friedrich, Niels Heise, Benjamin Brandes, Hans-Peter Deigner, Ahmed Al-Harrasi, René Csuk

**Affiliations:** 1 Organic Chemistry, Martin–Luther University Halle–Wittenberg, Kurt–Mothes, Str. 2, D-06120 Halle (Saale), Germany; sophie.hoenke@chemie.uni-halle.de (S.H.); martin.christoph@student.uni-halle.de (M.A.C.); sander.friedrich@student.uni-halle.de (S.F.); niels.heise@student.uni-halle.de (N.H.); Benjamin.brandes@chemie.uni-halle.de (B.B.); 2 Institute of Precision Medicine, Medical and Life Science Faculty, Furtwangen University, Jakob–Kienzle–Str. 17, D-78054 Villigen–Schwenningen, Germany; dei@hs-furtwangen.de; 3 Chair of Oman’s Medicinal Plants and Marine Natural Products, University of Nizwa, P.O. Box 33, Birkat Al-Mauz, PC 616 Nizwa, Oman; aharrasi@unizwa.edu.om

**Keywords:** oleanolic acid, ursolic acid, betulinic acid, platanic acid, 1,4-cyclohexyldiamines, cytotoxicity

## Abstract

Pentacyclic triterpenoids oleanolic acid, ursolic acid, betulinic acid, and platanic acid were acetylated and converted into several amides **9**–**31**; the cytotoxicity of which has been determined in sulforhodamine B assays employing seral human tumor cell lines and nonmalignant fibroblasts. Thereby, a betulinic acid/*trans*-1,4-cyclohexyldiamine amide showed excellent cytotoxicity (for example, EC_50_ = 0.6 μM for HT29 colon adenocarcinoma cells).

## 1. Introduction

Pentacyclic triterpenes represent an important class of secondary natural products [1,2,3,4,5,6,7,8,9,10]. Their research began very early in the history of chemistry: for example, J. T. Lowitz’ betulin was the first to be extracted from plant material in pure form and described in its physicochemical properties as early as 1788 [11]. Great progress in their discovery, isolation, and especially in the elucidation of their complex structures was made at the beginning and in the middle of the 20th century. In this period, the first partial syntheses also took place. Their pharmaceutical/medical potential, however, was only recognized much later. The observation of E. Pisha in 1995 describing, for the first time, the cytotoxic effect of betulinic acid on melanoma cells was groundbreaking in this respect [12]. Since this observation, the research of this class of natural substances has intensified tremendously.

**Figure 1:**
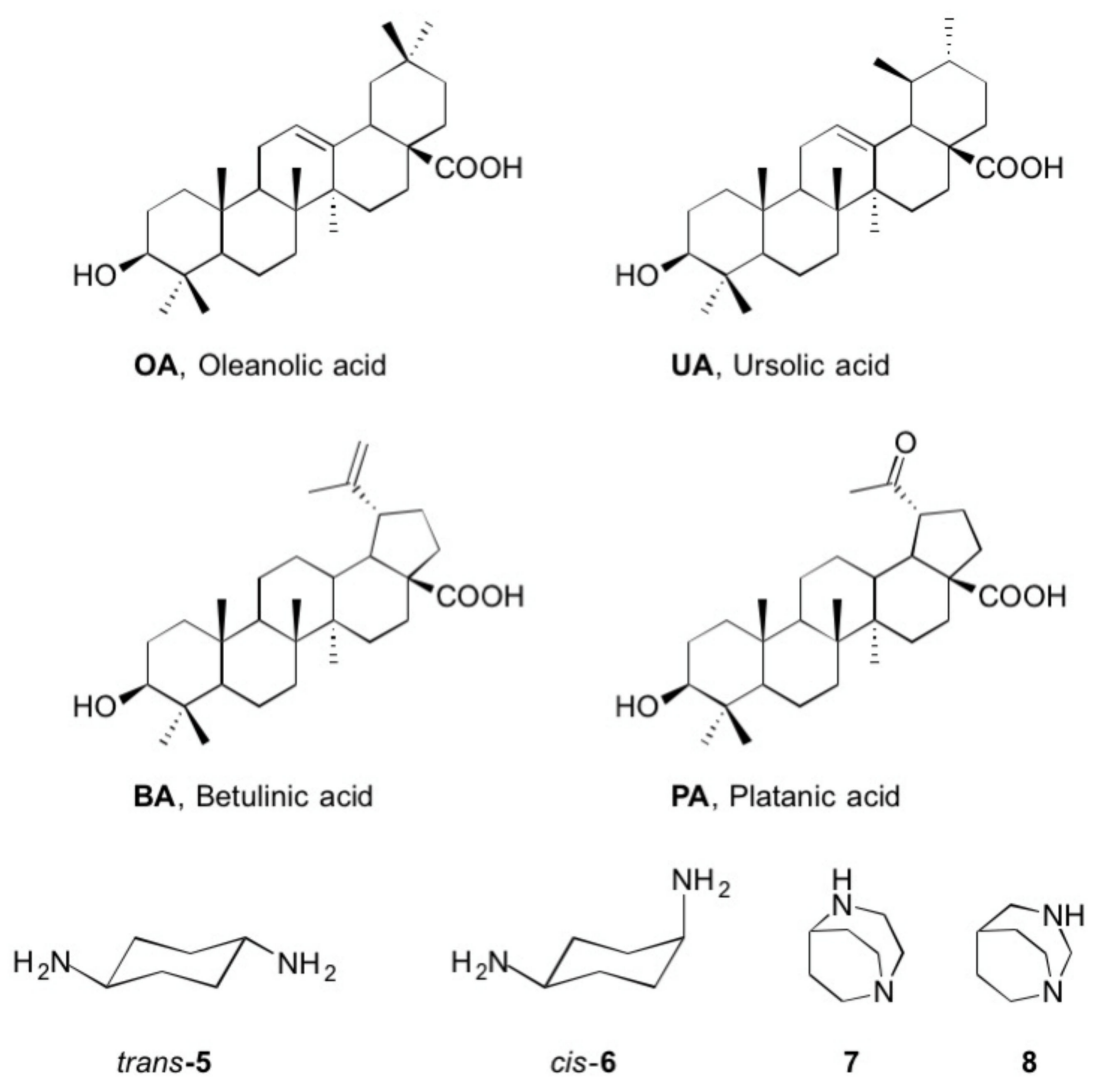
Structures of triterpenoic acids **OA**, **UA**, **BA**, and **PA** as well as of trans- and cis-1,4-cylohexyldiamines **5** and **6**, as well as bicyclic diamines **7** and **8**, respectively.

While unsubstituted triterpene carboxylic acids, e.g., oleanolic acid (**OA**) [13,14,15], ursolic acid (**UA**) [6], betulinic acid (**BA**) [16], and platanic acid [17,18,19,20] (**PA**) (Figure 1) have a relatively low cytotoxicity, the 3-*O*-acetylated amides of these compounds have especially become the focus of scientific interest in recent years. For example, triterpenoic benzyl amides (such as EM2) [21,22] and (homo)-piperazinyl-amides [23,24], as well as the rhodamine B conjugates [24] of the latter, have cytotoxic effects on numerous human tumor cell lines even in nanomolar concentrations [25]. However, some of these mitocanic compounds are quite cytotoxic to nonmalignant cells, and selective cytotoxicity can only be achieved by a several well-defined conjugates [25].

To get a deeper insight in the importance and role of a diamine-derived spacer, two different types of diamines (Figure 1) were selected. Both types have never been used in the context of triterpenes. On the one hand we selected 1,4-cyclohexyldiamines **5** and **6**, and on the other hand 1,4- (or 1,3)-diazabicyclo[3.2.2]nonanes **7** and **8**, respectively.

Triterpenoic amides holding both a piperazinyl or homopiperazinyl moiety and an extra rhodamine B moiety were moderately to highly cytotoxic to a variety of human tumor cell lines [25]. A somewhat differentiated picture, however, was found for compounds holding an ethylenediamine moiety instead [24]. Several of these compounds were cytotoxic while other analogs were not [17,18,21,26]. To evaluate the influence of a moiety being more rigid than ethylenediamine but as bulky as piperazine, we came across 1,4-cyclohexyldiamines **5** and **6**. The skeleton shows some similarity to piperazine concerning its flexibility and the conformation of the ring but also holds two primary amino groups, thus resembling ethylenediamine.

Since the rigidity of the diamine attached to the essential carboxyl group of the triterpenoid backbone might influence the cytotoxicity of the compounds, 1,4-diazabicyclo[3.2.2] nonane (**7**) [27] and 1,3-diazabicyclo[3.2.2]nonane (**8**) [28] were selected as amine components, too. Due to their structural similarity to a (homo)-piperazinyl spacer aside from holding an additional chain between the nitrogen, this would result in an increased rigidity of the molecule.

## 2. Results

**Scheme 1:**
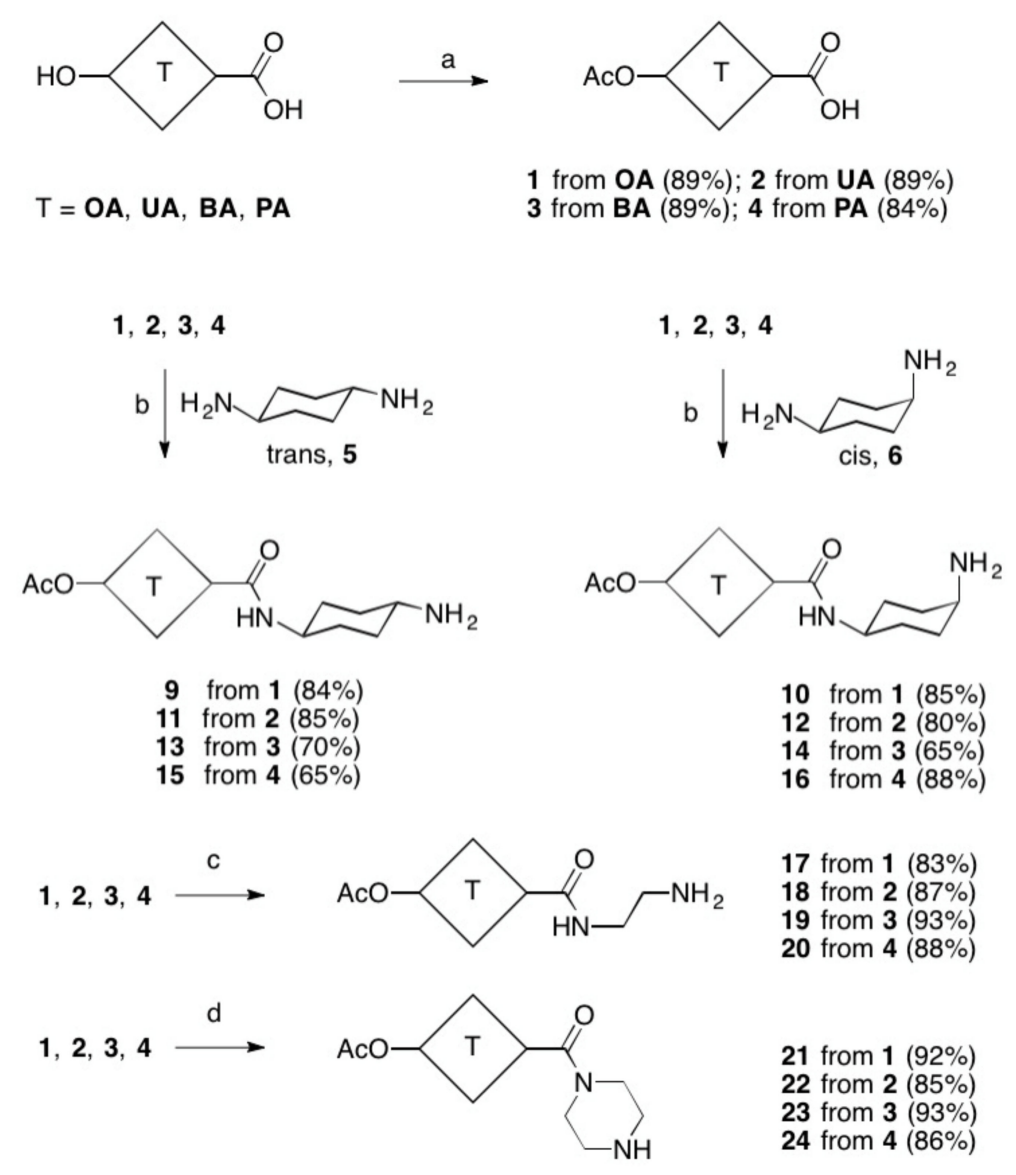
Reactions and conditions: (**a**) Ac_2_O, DCM, TEA, DMAP (cat.), 20 °C, 1d; (**b**) DCM, DMF (cat.), (COCl)_2_, then **5** or **6**, TEA, DMAP (cat.), 23 °C, 1d; (**c**) DCM, DMF (cat.), (COCl)_2_, then H_2_N-(CH_2_)_2_-NH_2_, TEA, DMAP (cat.), 23 °C, 1d; and (**d**) DCM, DMF (cat.), (COCl)_2_, then piperazine, TEA, DMAP (cat.), 23 °C, 1d.

The 1,4-dicyclohexyldiamine scaffold exists in two different stereoisomers holding either a *cis*- or a *trans*-configuration of the amino groups. Additionally, both compounds are *meso*-compounds. For the synthesis of the target compounds, triterpenoic acids oleanolic acid (**OA**), ursolic acid (**UA**), betulinic acid (**BA**), and platanic acid (**PA**) (Figure 1) were acetylated yielding well-known 3-*O*-acetyl compounds **1**–**4**, respectively. After having been activated with oxalyl chloride *trans*-1,4-cyclohexyldiamine (**5**) or *cis*-1,4-dicyclohexyldiamine (**6**) was added, and final target compounds **9**–**16** were obtained in yields between 65–85% (Scheme 1).

**Scheme 2:**
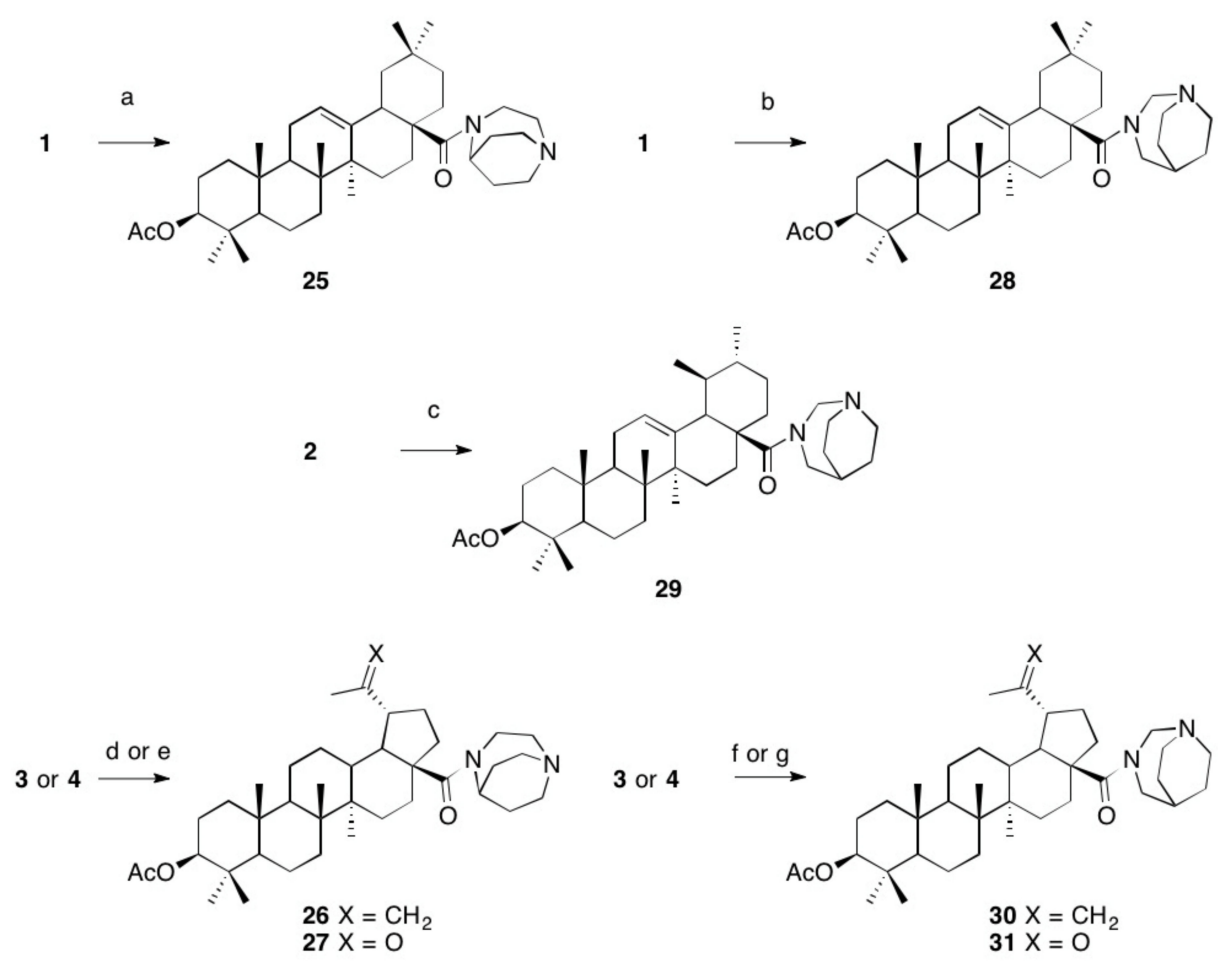
Reactions and conditions: (**a**) DCM, DMF (cat.), (COCl)_2_, then **7**, TEA, DMAP (cat.), 23 °C, 1d, 73%; (**b**) DCM, DMF (cat.), (COCl)_2_, then **8**, TEA, DMAP (cat.), 23 °C, 1d, 73%; (**c**) DCM, DMF (cat.), (COCl)_2_, then **8**, TEA, DMAP (cat.), 23 °C, 1d, 73%; (**d**) or (**e**) from **3**: DCM, DMF (cat.), (COCl)_2_, then **7**, TEA, DMAP (cat.), 23 °C, 1d, 74%, from **4**: 99%; (**f**) or (**g**) from **3** and **8**: 95%; from **4** and **8**: 73%.

The compounds are characterized, as exemplified for **OA** derived *trans*-**9** and *cis*-**10** in their ^13^C NMR spectra holding a signal for C-28 (amide) at δ = 179.8 ppm. The synthesis of the two amines **7** and **8** started from commercially available quinuclidin-3-one hydrochloride as previously reported [27,28]. Acetates **1**–**4** were activated with oxalyl chloride followed by the addition of either ethylenediamine (→ products **17**–**20**, Scheme 1), piperazine (→ products **21**–**24**, Scheme 1), **7** (→ products **25**–**27**, Scheme 2), or **8** (→ products **28**–**31**, Scheme 2).

For cytotoxicity screening, parent triterpenoic acid, acetates **1**–**4** and products **9**–**31** were subjected to sulforhodamine B assays (SRB) employing several human tumor cell lines as well as nonmalignant fibroblasts (NIH 3T3). The results from these assays are summarized in Table 1, Table 2 and Table 3.

As can be seen from Table 1, Table 2 and Table 3, there is, by and large, no significant difference in the cytotoxicity of the individual compounds; the cytotoxic activity is independent of the used spacer. Betulinic acid (**BA**)-derived derivatives are slightly more cytotoxic than the other derivatives. The selectivity between malignant cells and the nonmalignant fibroblast cell line NIH 3T3 is practically not affected by the choice of different spacers. The better cytotoxicity of the 3-*O*-acetylated pentacyclic triterpenoids as compared to their analogues holding an unprotected hydroxyl moiety at position C-3 might be explained by a better bioavailability of the former. The better cytotoxicity of derivatives derived from **BA** as compared to those from **PA**, however, cannot be explained but seems to be quite general inasmuch as the parent compound **BA** is more cytotoxic and PA, and the same also holds true for the corresponding 3-*O*-acetates. Comparison of the cytotoxicity of compounds **15** and **16** would have been interesting but failed because of the insolubility of **16** under the conditions of the assay. This might also have been caused by the presence of a weak hydrogen bond between the axially arranged amino group in **16** with the amide group. This assumption is also confirmed by the fact that **16** has a higher melting point than **15**, and it can be assumed that solvation leads to an additional bias against the axial position.

All compounds show only low selectivity. This finding distinguishes these compounds from substituted benzylamides (such as EM2) [21] but also from derivatives with an additional rhodamine B [24,25] moiety being present.

## 3. Conclusions

Pentacyclic triterpenoids oleanolic acid, ursolic acid, betulinic acid, and platanic acid were acetylated. These acetates were treated with oxalyl chloride followed by the addition of ethylenediamine or several monocyclic and bicyclic diamines to provide amides **9**–**31**, whose cytotoxicity has been determined in sulforhodamine B assays employing seral human tumor cell lines and nonmalignant fibroblasts. Thereby, a betulinic acid/trans 1,4-cyclohexyldiamine amide showed excellent cytotoxicity (for example, EC_50_ = 0.6 μM for HT29 colon adenocarcinoma cells), but the selectivity tumor cell/nontumor cell could not be improved. This finding distinguishes these compounds from previously investigated substituted benzylamides but also from piperazinyl-rhodamine B conjugates.

## 4. Experimental

NMR spectra were recorded using the Varian spectrometers (Darmstadt, Germany) DD2 and VNMRS (400 and 500 MHz, respectively). MS spectra were taken on an Advion expression^L^ CMS mass spectrometer (Ithaca, USA; positive ion polarity mode, solvent: methanol, solvent flow: 0.2 mL/min, spray voltage: 5.17 kV, source voltage: 77 V, APCI corona discharge: 4.2 μA, capillary temperature: 250 °C, capillary voltage: 180 V, sheath gas: N_2_). Thin-layer chromatography was performed on precoated silica gel plates supplied by Macherey-Nagel (Düren, Germany). IR spectra were recorded on a Spectrum 1000 FT-IR-spectrometer from Perkin Elmer (Rodgau, Germany). The UV/Vis-spectra were recorded on a Lambda 14 spectrometer from Perkin Elmer (Rodgau, Germany). The melting points were determined using the Leica hot-stage microscope Galen III (Leica Biosystems, Nussloch, Germany) and are uncorrected. The solvents were dried according to usual procedures. The triterpenoic acids were bought from “Betulinines” (Stříbrná Skalice, Czech Republic) and used as received.

### 4.1. Cell Lines and Culture Conditions

Following human cancer cell lines A375 (malignant melanoma), HT29 (colon adenocarcinoma), MCF-7 (breast cancer), A2780 (ovarian carcinoma), FaDu (pharynx carcinoma), and nonmalignant mouse fibroblasts NIH 3T3 were used. All cell lines were obtained from the Department of Oncology (Martin Luther University Halle-Wittenberg). Cultures were maintained as monolayers in RPMI 1640 medium with l-glutamine (Capricorn Scientific GmbH, Ebsdorfergrund, Germany) supplemented with 10% heat-inactivated fetal bovine serum (Sigma-Aldrich GmbH, Steinheim, Germany) and 1% penicillin/streptomycin (Capricorn Scientific GmbH, Ebsdorfergrund, Germany) at 37 °C in a humidified atmosphere with 5% CO_2_.

### 4.2. Cytotoxicity Assay (SRB Assay)

For the evaluation of the cytotoxicity of the compounds the sulforhodamine-B (Kiton-Red S, ABCR GmbH, Karlsruhe, Germany), a microculture colorimetric assay was used as previously reported. The EC_50_ values were averaged from three independent experiments performed each in triplicate calculated from semilogarithmic dose-response curves applying a nonlinear 4P Hills-slope equation. In short, cells were seeded into 96 well plates at day 0 at appropriate cell densities to prevent confluence of the cells during the period of the experiment. After 24 h, the cells were treated with different concentrations (1, 3, 7, 12, 20, and 30 μM), but the final concentration of DMSO/DMF never exceeded 0.5%, which was nontoxic to the cells. After 72 h treatment, the supernatant media from the 96 well plates were discarded, and then the cells were fixed with 10% trichloroacetic acid and allowed to rest at 4 °C. After 24 h of fixation, the cells were washed in a strip washer and then dyed with SRB solution (200 μL, 10 mM) for 20 min. Then, the plates were washed four times with 1% acetic acid to remove the excess of the dye and allowed to air-dry overnight. Tris base solution (200 μL, 10 mM) was added to each well. The absorbance was measured with a 96 well plate reader from Tecan Spectra.

### 4.3. General Procedure for the Synthesis of Acetates ***1***–***4*** (GPA)

To a solution of the triterpenoic acid (**OA**, **UA**, **BA**, **PA**, 1 eq.) in dry DCM, acetic anhydride (3 eq.), triethylamine (3 eq.) and DMAP (cat.) were added, and stirring at 20 °C was continued for 1 day. Usual aqueous workup followed by recrystallization from ethanol furnished products **1**–**4**.

### 4.4. General Procedure for the Synthesis of Amides ***9***–***31*** (GPB)

To the solution of the acetylated triterpenoic acid (**1**–**4**, 1 eq.) in dry DCM, a drop of dry DMF and oxalyl chloride (4 eq.) were added at 0 °C. Stirring at 25 °C was continued until the evolution of gases had ceased. The volatiles were removed under reduced pressure. The corresponding amine (3 eq.) was dissolved in dry DCM (20 mL), and a solution of TEA (4.2 eq.), DMAP (cat.) in dry DCM (10 mL) was added. To this mixture, the reaction mixture (dissolved in dry DCM) from above was slowly added at 0 °C, and stirring at 23 °C was continued for 1 day. The usual aqueous workup followed by liquid column chromatography (CHCl_3_/MeOH) gave the products **9**–**31**, respectively.

*3β-Acetyloxy-olean-12-en-28-oic acid* (**1**). Following GPA, compound **1** (4.89 g, 89%) was obtained as a colorless solid; R_f_ = 0.54 (hexanes/ethyl acetate, 3:1); m.p.: 259–263 °C (lit.: [29] 266–268 °C); $\alpha_{D}^{20}$ = +74.1° (c 0.43, CHCl_3_) [lit.: [29] $\alpha_{D}^{20}$ = +74.0° (c 1, CHCl_3_)]; MS (ESI, MeOH): m/z 499.1 ([M + H]^+^, 9), 521.3 (38%, [M + Na]^+^), 1019.4 (100%, [2M + Na]^+^).
*3β-Acetoxy-urs-12-en-28-oic acid* (**2**). Following GPA, compound **2** (4.89 g, 89%) was obtained as a colorless solid; R_f_ = 0.71 (toluene/ethyl acetate/heptane/formic acid, 80:26:10:5); m.p.: 287–290 °C (lit.: [30] 289–290 °C); $\alpha_{D}^{20}$ = +68.9° (c 0.315, CHCl_3_) [lit.: [31] $\alpha_{D}^{20}$ = +72.3° (c 0.5, CHCl_3_)]; MS (ESI, MeOH): m/z 499.0 ([M + H]^+^, 74), 516.3 (36%, [M + NH_4_]^+^), 521.5 (34%, [M + Na]^+^);
*3β-Acetoxy-lup-20(29)-en-28-oic acid* (**3**). Following GPA, compound **3** (4.90 g, 89%) was obtained as a colorless solid; R_f_ = 0.58 (hexanes/ethyl acetate, 4:1); m.p.: 281–283 °C (lit.: [32] 280–282 °C); $\alpha_{D}^{20}$ = +25.6° (c 0.35, CHCl_3_) [lit.: [33] $\alpha_{D}^{20}$ = +26.4° (c 0.54, CHCl_3_)]; MS (ESI, MeOH): m/z 487.1 (28%, [M− H]^−^) 995.3 (100%, [2M − H]^−^), 1018.2 (28%, [2M − 2H + Na]^−^).
*3β-Acetoxy-20-oxo-30-norlupan-28-oic acid* (**4**). Following GPA, compound **4** (13.8 g, 84%) was obtained as a colorless solid; R_f_ = 0.50 (toluene/ethyl acetate/heptane/formic acid, 80:26:10:5); m.p.: 268–270 °C (decomp.), (lit.: [34] 252–255 °C); $\alpha_{D}^{20}$ = −9.1° (c 0.34, CHCl_3_) [lit.: [34] $\alpha_{D}^{20}$ = −9.5° (c 0.8, CHCl_3_)]; MS (ESI, MeOH): m/z 999.3 (100%, [2M − H]^−^).
*Trans-cyclohexyl-1,4-diamine* (**5**) *and cis-cyclohexyl-1,4-diamine* (**6**). These compounds were commercially obtained from Merck and used as received.
*1,4-Diazabicyclo[3.2.2]nonane* (**7**) and *1,3-diazabicyclo[3.2.2]nonane* (**8**). These compounds were prepared from quinuclidin-3-one hydrochloride as previously reported [27,28].
*(3β)-28-[(trans-4-Aminocyclohexyl)amino]-28-oxoolean-12-en-3-yl acetate* (**9**). Following GPB, compound **9** (1.01 g, 84%) was obtained as a colorless solid; R_f_ = 0.66 (CHCl_3_/MeOH, 8:2); m.p.: 203–205 °C (decomp.); $\alpha_{D}^{20}$ = +23.6° (c 0.35, MeOH); IR (ATR): ν = 3423 w, 2945 m, 1703 m, 1618 m, 1430 s, 1332 s, 1036 s, 817 s. 749 s cm^−1^; ^1^H NMR (500 MHz, CD_3_OD): δ = 6.64 (m, 1H, NH), 5.43 (t, J = 3.6 Hz, 1H, 12-H), 4.48 (dd, J = 11.2, 4.9 Hz, 1H, 3-H), 3.89 (s, 1H, 33-H), 3.35–3.32 (m, 1H, 36-H), 2.85–2.79 (m, 1H, 18-H), 2.20–2.07 (m, 1H, 16-H_a_), 2.05 (s, 3H, 32-H), 1.99–1.28 (m, 25H, 34-H, 35-H, 37-H, 38-H, 1-H_b_, 22-H, 2-H, 15-H_a_, 6-H, 11-H, 7-H, 1-H_a_, 21-H, 9-H, 19-H_b_), 1.23 (s, 3H, 27-H), 1.22–1.02 (m, 3H, 15-H_b_, 16-H_b_, 19-H_a_), 1.00 (s, 3H, 25-H), 0.98 (s, 3H, 29-H), 0.94 (s, 3H, 30-H), 0.91 (s, 3H, 24-H), 0.91 (s, 3H, 23-H), 0.89 (m, 1H, 5-H), 0.84 (s, 3H, 26-H) ppm; ^13^C NMR (126 MHz, CD_3_OD): δ = 179.8 (C-28), 145.5 (C-13), 123.9 (C-12), 82.4 (C-3), 56.6 (C-5), 49.9 (C-9), 49.5 (C-33), 47.7 (C-19), 47.5 (C-17), 46.3 (C-36), 43.8 (C-18), 43.1 (C-14), 40.7 (C-8), 39.3 (C-1), 38.7 (C-4), 38.1 (C-10), 35.1 (C-21), 34.2 (C-34, C-38), 33.8 (C-35, C-37), 33.5 (C-30), 31.6 (C-20), 28.5 (C-15), 28.5 (C-23), 28.1 (C-7), 27.8 (C-22), 26.3 (C-27), 24.5 (C-2), 24.5 (C-11), 24.0 (C-16), 23.9 (C-29), 21.1 (C-32), 19.3 (C-6), 18.1 (C-26), 17.1 (C-24), 15.9 (C-25) ppm; ESI, MeOH): m/z 595.4 (100%, [M + H]^+^), 1189.3 (5%, [2M + H]^+^); analysis calcd for C_38_H_62_N_3_O_4_ (594.91): C 76.72, H 10.50, N 4.71; found: C 76.49, H 10.71, N 4.55. Please see Supplementary Materials.
*(3β)-28-[(cis-4-Aminocyclohexyl)amino]-28-oxoolean-12-en-3-yl acetate* (**10**). Following GPB, compound **10** (1.02 g, 85%) was obtained as a colorless solid; R_f_ = 0.673 (CHCl_3_/MeOH, 8:2); m.p.: 197–200 °C (decomposition); $\alpha_{D}^{20}$ = +3.8° (c 0.14, CHCl_3_); IR (ATR): ν = 3406 w, 2944 m, 1704 m, 1621 m, 1524 s, 1428 s, 1313 s, 1099 m, 1028 s, 821 s, 763 m cm^−1^; ^1^H NMR (500 MHz, CD_3_OD): δ = 6.61–6.55 (m, 1H, NH), 5.37 (t, 1H, 12-H), 4.42 (d, 1H, 3-H), 3.60–3.52 (m, 1H, 33-H), 3.29–3.24 (m, 1H, 36-H), 2.10–1.93 (m, 1H, 18-H), 1.99 (s, 3H, 32-H), 1.93–1.85 (m, 8H, 34-H, 35-H, 37-H, 38-H), 1.85–1.21 (m, 14H, 1-H_b_, 22-H_a_, 2-H, 16-H_a_, 15-H_b_, 6-H, 11-H_b_, 7-H_b_, 1-H_a_, 21-H_b_, 9-H, 19-H_b_), 1.16 (s, 3H, 27-H), 1.16–0.97 (m, 5H, 15-H_a_,16-H_b_,19-H_a_, 21-H_a_, 22-H_b_), 0.94 (s, 3H, 26-H), 0.91 (s, 3H, 24-H), 0.88 (s, 3H, 25-H), 0.85 (s, 3H, 29-H), 0.84 (s, 3H, 30-H), 0.83–0.81 (m, 1H, 5-H), 0.78 (s, 3H, 23-H) ppm; ^13^C NMR (126 MHz, CDCl_3_): δ = 179.8 (C-28), 172.8 (C-31), 145.4 (C-13), 123.9 (C-12), 82.4 (C-3), 56.6 (C-5), 49.8 (C-9), 49.5 (C-33), 47.6 (C-19), 46.2 (C-17), 43.7 (C-18), 43.1 (C-14), 42.8 (C-36), 40.6 (C-21), 39.2 (C-8), 38.6 (C-4), 38.0 (C-1), 37.0 (C-10), 34.2 (C-7), 33.8 (C-34, C-38), 33.5 (C-29, C-30), 31.5 (C-35, C-37), 28.0 (C-20), 27.7 (C-22), 27.3 (C-15), 27.2 (C-16), 26.3 (C-27), 24.5 (C-2), 21.1 (C-32), 19.2 (C-6), 18.3 (C-23), 18.1 (C-26), 17.1 (C-24), 15.9 (C-25) ppm; MS (ESI, MeOH): m/z 595.4 (100%, [M + H]^+^), 1190.4 (8%, [2M + H]^+^); analysis calcd for C_38_H_62_N_3_O_4_ (594.91): C 76.72, H 10.50, N 4.71; found: C 76.59, H 10.75, N 4.46.
*(3β)-28-[(trans-4-Aminocyclohexyl)amino]-28-oxoursan-12-en-3-yl acetate* (**11**). Following GPB, compound **11** (1.02 g, 85%) was obtained as a colorless solid; R_f_ = 0.66 (CHCl_3_/MeOH, 8:2); m.p.: 189–193 °C (decomp.); $\alpha_{D}^{20}$ = +34.4° (c 0.31, MeOH); IR (ATR): ν = 3420 w, 2928 m, 1734 m, 1621 m, 1523 m,1314 s, 1244 s, 1096 s, 1027 s, 985 m, 902 m, 822 s cm^−1^; ^1^H NMR (500 MHz, CD_3_OD): δ = 6.90 (m, 1H, NH), 5.28 (t, J = 3.7 Hz, 1H, 12-H), 4.42 (m, 1H, 3-H), 3.64–3.51 (m, 1H, 33-H), 3.10–2.98 (m, 1H, 36-H), 2.16–2.12 (m, 1H, 18-H), 2.07–1.97 (m, 3H, 16-H_b_, 35-H_a_, 37-H_a_), 1.99 (s, 3H, 30), 1.94–1.87 (m, 3H, 11, 21-H_a_), 1.84–1.72 (m, 3H, 15-H_b_, 34-H_b_, 38-H_b_), 1.69–1.26 (m, 17H, 1-H_b_, 2-H, 6-H, 7-H, 9-H, 16-H_a_, 19-H, 22-H, 34-H_a_, 35-H_b_, 37-H_b_, 38-H_a_), 1.10 (s, 3H, 27-H), 1.07–0.99 (m, 3H, 1-H_a_, 15-H_a_, 20-H), 0.95 (s, 3H, 25-H), 0.93 (s, 3H, 32-H), 0.87 (s, 3H, 26-H), 0.85 (s, 3H, 24-H), 0.84 (s, 3H, 23-H), 0.82–0.80 (m, 1H, 5-H), 0.79 (s, 3H, 29-H) ppm; ^13^C NMR (126 MHz, CD_3_OD): δ = 179.5 (C-28), 172.8 (C-31), 139.9 (C-13), 126.8 (C-12), 82.4 (C-3), 56.7 (C-5), 53.9 (C-18), 50.6 (C-36), 49.9 (C-9), 48.8 (C-17), 48.7 (C-33), 43.4 (C-14), 40.8 (C-19), 40.2 (C-20), 39.4 (C-1), 38.7 (C-8), 38.0 (C-4), 34.2 (C-10), 31.9 (C-7), 31.1 (C-21), 30.7 (C-38, C-34), 30.5 (C-37, C-35), 28.9 (C-15), 28.6 (C-23), 24.9 (C-16), 24.5 (C-2), 24.4 (C-11), 24.1 (C-27), 21.6 (C-32), 21.1 (C-30), 19.3 (C-6), 18.2 (C-29), 17.2 (C-24), 16.0 (C-25) ppm; MS (ESI, MeOH): m/z 595.4 (100%, [M + H]^+^), 1211.6 (4%, [2M + Na]^+^); analysis calcd for C_38_H_62_N_3_O_4_ (594.91): C 76.72, H 10.50, N 4.71; found: C 76.60, H 10.83, N 4.52.
*(3β)-28-[(Cis-4-Aminocyclohexyl)Amino]-28-Oxoursan-12-En-3-Yl Acetate* (**12**). Following GPB, compound **12** (0.96 g, 80%) was obtained as a colorless solid; R_f_ = 0.67 (CHCl_3_/MeOH, 8:2); m.p.: 186–190 °C (decomp.); $\alpha_{D}^{20}$ = +26.7° (c 0.10, CHCl_3_); IR (ATR): ν = 3416 m, 2929 m, 1625 m, 1520 m, 1326 s, 1245 s, 1028 s, 823 m cm^−1^; ^1^H NMR (500 MHz, CD_3_OD): δ = 6.52 (m, 1H, NH), 5.36 (t, J = 3.6 Hz, 1H, 12-H), 4.42 (dd, J = 11.0, 5.0 Hz, 1H, 3-H), 3.61–3.51 (m, 1H, 33-H), 3.28–3.26 (m, 1H, 36-H), 2.12–2.05 (m, 1H, 18-H), 1.99 (s, 3H, 30-H), 2.06–1.96 (m, 3H, 16-H_b_, 35-H_a_, 37-H_a_), 1.96–1.89 (m, 3H, 11-H, 21-H_a_), 1.89–1.75 (m, 3H, 15-H_b_, 34-H_b_, 38-H_b_), 1.74–1.27 (m, 16H, 34-H_a_, 35-H_b_, 37-H_b_,38-H_a_, 1-H_b_, 22-H, 2-H, 16-H, 6-H, 9-H, 7-H), 1.12 (s, 3H, 27-H), 1.08–1.00 (m, 3H, 1-H_a_, 15-H_a_, 20-H), 0.95 (s, 3H, 25-H), 0.94 (s, 3H, 32-H), 0.98 (m, 3H, 26-H), 0.85 (s, 3H, 24-H), 0.84 (s, 3H, 23-H), 0.83–0.81 (m, 1H, 5-H), 0.79 (s, 3H, 29-H) ppm; ^13^C NMR (126 MHz, CD_3_OD): δ = 179.7 (C-28), 172.8 (C-31), 140.4 (C-13), 127.0 (C-12), 82.3 (C-3), 56.6 (C-5), 54.6 (C-18), 49.8 (C-9), 49.6 (C-36), 49.0 (C-17), 48.8 (C-33), 43.5 (C-14), 40.9 (C-19), 40.0 (C-20), 39.4 (C-1), 38.7 (C-8), 38.0 (C-4), 34.1 (C-22), 31.9 (C-7), 29.0 (C-15), 28.6 (C-23), 28.0 (C-21), 27.1 (C-34, C-38), 27.0 (C-35, C-37), 25.2 (C-16), 24.5 (C-2), 24.4 (C-11), 23.9 (C-27), 21.5 (C-32), 21.1 (C-30), 19.2 (C-6), 18.1 (C-29), 17.6 (C-26), 17.2 (C-24), 16.1 (C-25) ppm; MS (ESI, MeOH): m/z 595.4 (100%, [M + H]^+^), 1189.4 (10%, [2M + H]^+^); analysis calcd for C_38_H_62_N_3_O_4_ (594.91): C 76.72, H 10.50, N 4.71; found: C 76.54, H 10.69, N 4.48.
*(3β)-28-[(trans-4-Aminocyclohexyl)amino]-28-oxolup-20(29)-en-3-yl acetate* (**13**). Following GPB, compound **13** (0.42 g, 70%) was obtained as a colorless solid; R_f_ = 0.595 (CHCl_3_/MeOH, 8:2); m.p.: 205–212 °C (decomp.); $\alpha_{D}^{20}$ = +0.1° (c 0.17, MeOH); IR (ATR): ν = 2940 m, 1731 m, 1637 m, 1513 m, 1369 m, 1244 s, 1026 m, 978 m, 882 m, 751 s cm^−1^; ^1^H NMR (500 MHz, CDCl_3_): δ = 4.71 (s, 1H, 29-H_a_), 4.57 (s, 1H, 29-H_b_), 4.49–4.41 (m, 1H, 3-H), 3.96–3.80 (m, 1H, 36-H), 3.76–3.63 (m, 1H, 33-H), 3.22–3.03 (m, 1H, 19-H), 2.40 (td, J = 12.3, 3.6 Hz, 1H, 13), 2.02 (s, 3H, 32-H), 1.98–1.02 (m, 28H, 37-H, 35-H, 38-H, 34-H, 1-H_a_, 22-H_a_, 12-H_a_, 2-H, 18-H, 16-H, 15-H_a_, 6-H, 11-H, 7-H, 1-H_b_, 21-H_a_, 9-H, 15-H_b_), 1.66 (s, 3H, 30-H), 1.01–0.96 (m, 2H, 1-H_b_, 12-H_b_), 0.94 (s, 3H, 27-H), 0.92 (s, 3H, 26-H), 0.82 (d, J = 1.5 Hz, 6H, 23-H, 24-H), 0.81 (s, 3H, 25-H), 0.78–0.73 (m, 1H, 5-H) ppm; ^13^C NMR (126 MHz, CDCl_3_): δ = 175.6 (C-28), 171.1 (C-31), 151.1 (C-20), 109.5 (C-29), 81.1 (C-3), 55.6 (C-17), 55.6 (C-5), 50.7 (C-9), 50.3 (C-18), 50.1 (C-33), 47.4 (C-19), 47.0 (C-36), 42.6 (C-14), 40.9 (C-8), 39.2 (C-13), 38.5 (C-22), 38.5 (C-1), 37.9 (C-10), 37.2 (C-4), 34.4 (C-7), 33.9 (C-16), 33.8 (C-34, C-38), 31.5 (C-35, C-37), 31.0 (C-21), 29.5 (C-15), 28.1 (C-23), 25.7 (C-12), 23.8 (C-2), 21.4 (C-32), 21.1 (C-11), 19.6 (C-30), 18.3 (C-6), 16.6 (C-24), 16.4 (C-25), 16.3 (C-26), 14.7 (C-27) ppm; MS (ESI, MeOH/CHCl_3_, 4:1): m/z 593.3 (100%, [M − H]^−^), 629.3 (80%, [M + Cl]^−^); analysis calcd for C_38_H_62_N_3_O_4_ (594.91): C 76.72, H 10.50, N 4.71; found: C 76.47, H 10.89, N 4.43.
*(3β)-28-[(Cis-4-Aminocyclohexyl)Amino]-28-Oxolup-20(29)-En-3-Yl Acetate* (**14**). Following GPB, compound **14** (0.39 g, 65%) was obtained as a colorless solid; R_f_ = 0.634 (CHCl_3_/MeOH, 8:2); m.p.: 230–235 °C (decomp.); $\alpha_{D}^{20}$ = +9.7° (c 0.19, MeOH); IR (ATR): ν = 2940 s, 1731 m, 1620 m, 1505 m, 1368 m, 1244 s, 1027 m, 978 m, 751 s cm^−1^; ^1^H NMR (500 MHz, CDCl_3_): δ = 4.73 (s, 1H, 29-H_a_), 4.59 (s, 1H, 29-H_b_), 4.46 (dd, J = 10.3, 5.7 Hz, 1H, 3-H), 4.00 (s, 1H, 36-H), 3.47 (s, 1H, 33-H), 3.10 (td, J = 11.0, 3.9 Hz, 1H, 19-H), 2.46 (dd, J = 11.7, 2.0 Hz, 1H, 13-H), 2.03 (s, 3H, 32-H), 2.02–1.05 (m, 28H, 37-H, 35-H, 38-H, 34-H, 1-H_a_, 22-H_a_, 12-H_a_, 2-H, 18-H, 16-H, 15-H_a_, 6-H, 11-H, 7-H, 1-H_b_, 21-H_a_, 9-H, 15-H_b_), 1.67 (s, 3H, 30-H), 1.03–0.96 (m, 2H, 1-H_a_, 12-H_b_), 0.95 (s, 3H, 27-H), 0.92 (s, 3H, 26-H), 0.84 (s, 6H, 23-H, 24-H), 0.83 (s, 3H, 25-H), 0.80–0.75 (m, 1H, 5-H) ppm; ^13^C NMR (126 MHz, CDCl_3_): δ = 175.6 (C-28), 171.0 (C-31), 150.8 (C-20),109.5 (C-29), 80.9 (C-3), 55.6 (C-17), 55.4 (C-5), 50.5 (C-9), 50.0 (C-18), 48.0 (C-33), 46.7 (C-19), 44.3 (C-36), 42.4 (C-14), 40.8 (C-8), 38.5 (C-22), 38.4 (C-1), 37.8 (C-10), 37.7 (C-13), 37.1 (C-4), 34.4 (C-7), 33.8 (C-16), 30.9 (C-21), 29.7 (C-34, C-38), 29.4 (C-15), 27.9 (C-23), 26.9 (C-35, C-37), 25.6 (C-12), 23.7 (C-2), 21.3 (C-32), 21.0 (C-11), 19.5 (C-30), 18.0 (C-6), 16.5 (C-24), 16.3 (C-26), 16.2 (C-25), 14.6 (C-27) ppm; MS (ESI, MeOH/CHCl_3_, 4:1): m/z 595.5 (100%, [M + H]^+^); analysis calcd for C_38_H_62_N_3_O_4_ (594.91): C 76.72, H 10.50, N 4.71; found: C 76.55, H 10.83, N 4.61.
*(3β)-28-[(trans-4-Aminocyclohexyl)amino]-20,28-dioxo-30-norlupan-3-yl acetate* (**15**). Following GPB, compound **15** (0.385 g, 65%) was obtained as a colorless solid; R_f_ = 0.595 (CHCl_3_/MeOH, 8:2); m.p.: 251–255 °C (decomp.); $\alpha_{D}^{20}$ = −17.0° (c 0.15, MeOH); IR (ATR): ν = 3384 w, 2941 s, 1710 m, 1633 m, 1516 m, 1368 m, 1025 m, 751 s cm^−1^; ^1^H NMR (500 MHz, CDCl_3_): δ = 4.49–4.41 (m, 1H, 3-H), 3.84–3.71 (m, 1H, 35-H), 3.46–3.27 (m, 1H, 19-H), 3.25–2.97 (m, 1H, 32-H), 2.33–2.18 (m, 1H, 13-H), 2.15 (s, 3H, 29-H), 2.07 (d, J = 15.8 Hz, 2H, 18-H, 21-H_a_), 2.03 (s, 3H, 31-H), 1.94–1.81 (m, 1H, 16-H_a_), 1.77–1.04 (m, 27-H, 36-H, 34-H, 37-H, 33-H, 22-H, 12-H, 2-H, 1-H_a_, 16-H_b_, 21-H_b_, 15-H_a_, 6-H, 11-H, 7-H, 9-H, 15-H_b_), 0.98 (s, 3H, 27-H), 0.96–0.92 (m, 1H, 1-H_b_), 0.90 (s, 3H, 26-H), 0.83–0.82 (m, 6H, 24-H, 25-H), 0.81 (s, 3H, 23-H), 0.80–0.76 (m, 1H, 5-H) ppm; ^13^C NMR (126 MHz, CDCl_3_): δ = 212.8 (C-20), 175.8 (C-28), 171.1 (C-30), 80.9 (C-3), 55.5 (C-5), 55.4 (C-17) 51.3 (C-19), 50.5 (C-9), 50.3 (C-33), 50.2 (C-18), 47.0 (C-36), 42.4 (C-14), 40.9 (C-8), 38.5 (C-1), 38.2 (C-22), 37.9 (C-10), 37.3 (C-4), 37.0 (C-13), 34.4 (C-7), 33.1 (C-16), 30.9 (C-33, C-37), 30.3 (C-29), 29.7 (C-36, C-34), 29.6 (C-15), 28.7 (C-21), 28.1 (C-23), 27.3 (C-12), 23.8 (C-2), 21.4 (C-31), 21.1 (C-11), 18.3 (C-6), 16.6 (C-24), 16.3 (C-25), 16.3 (C-26), 14.8 (C-27) ppm; MS (ESI, MeOH/CHCl_3_ 4:1): m/z 597.4 (100%, [M + 2H]^+^); analysis calcd for C_37_H_60_N_2_O_4_ (596.88): C 74.45, H 10.13, N 4.69; found: C 74.19, H 10.32, N 4.42.
*(3β)-28-[(cis-4-Aminocyclohexyl)amino]-20,28-dioxo-30-norlupan-3-yl acetate* (**16**). Following GPB, compound **16** (465 mg, 78%) was obtained as a colorless solid; R_f_ = 0.65 (CHCl_3_/MeOH, 8:2); m.p.: 257–260 °C (decomp.); $\alpha_{D}^{20}$ = −9.1° (c 0.14, CHCl_3_); IR (ATR): ν = 2936 m, 1729 m, 1600 s, 1517 m, 1369 m, 1245 s, 988 s, 804 m, 7451 m cm^−1^; ^1^H NMR (500 MHz, CDCl_3_): δ = 4.58–4.26 (m, 1H, 3-H), 4.00–3.83 (m, 1H, 35-H), 3.43 (dt, J = 11.4, 6.0 Hz, 1H, 19-H), 3.11–2.85 (m, 1H, 32-H), 2.21 (dt, J = 11.8, 4.5 Hz, 1H, 13-H), 2.14 (s, 3H, 29-H), 2.09–2.03 (m, 2H, 18-H, 21-H_a_), 2.01 (s, 3H, 31-H), 1.95–1.85 (m, 1H, 16-H_b_), 1.77–1.01 (m, 27-H, 36-H, 34-H, 37-H, 33-H, 22-H, 12-H, 2-H, 1-H_b_, 16-H_b_, 15-H, 21-H_b_, 6-H, 11-H, 7-H, 9-H), 0.97 (s, 3H, 27-H), 0.88 (s, 3H, 26-H), 0.87 (s, 1H, 1-H_a_), 0.82 (s, 3H, 25-H), 0.81 (s, 3H, 24-H), 0.80 (s, 3H, 23-H), 0.79–0.73 (m, 1H, 5-H) ppm; ^13^C NMR (126 MHz, CDCl_3_): δ = 213.0 (C-20), 175.3 (C-28), 171.0 (C-30), 81.0 (C-3), 55.5 (C-17), 55.4 (C-5), 51.3 (C-19), 50.5 (C-9), 50.2 (C-18), 47.6 (C-32), 45.1 (C-35), 42.3 (C-14), 40.8 (C-8), 38.5 (C-1), 38.2 (C-22), 37.9 (C-10), 37.2 (C-4), 36.9 (C-13), 34.3 (C-7), 33.2 (C-16), 30.8 (C-37, C-33), 30.4 (C-29), 29.6 (C-15), 28.7 (C-21), 28.0 (C-23), 27.9 (C-36, C-34), 27.6 (C-12), 23.8 (C-2), 21.4 (C-31), 21.1 (C-11), 18.3 (C-6), 16.6 (C-24), 16.3 (C-25), 14.8 (C-27) ppm; MS (ESI, MeOH/CHCl_3_, 4:1): m/z 597.2 (95%, [M − H]^−^), 631.3 (100%, [M + Cl]^−^); analysis calcd for C_37_H_60_N_2_O_4_ (596.88): C 74.45, H 10.13, N 4.69; found: C 74.23, H 10.39, N 4.37.
*(3β)-28-[(2-Aminoethyl)amino]-28-oxoolean-12-en-3-yl acetate* (**17**). This compound (0.69 g, 83%) was obtained from **1** following GPB as a colorless solid; [35,36] m.p. 211–214 °C (lit.: [36] 212–215 °C); $\alpha_{D}^{20}$ = +38.3° (c 0.4, CHCl_3_) [lit.: [36] $\alpha_{D}^{20}$ = +37.8° (c 0.35, CHCl_3_); MS (ESI, MeOH): m/z 541.2 (100%, [M + H]^+^).
*(3β)-28-[(2-Aminoethyl)amino]-28-oxours-12-en-3-yl acetate* (**18**). This compound (0.81 g, 87%) was obtained from **2** following GPB as a colorless solid; [37,38,39,40] m.p. 202–205 °C (lit.: [37] 140–142 °C); $\alpha_{D}^{20}$ = +39.0° (c 0.2, CHCl_3_) [lit.: [18] $\alpha_{D}^{20}$ = +39.4° (c 0.555, CHCl_3_); MS (ESI, MeOH): m/z 541.3 (100%, [M + H]^+^).
*(3β)-28-[(2-Aminoethyl)amino]-28-oxolup-20(29)-en-3-yl acetate* (**19**). This compound (0.86 g, 93%) was obtained from **3** following GPB as a colorless solid; [41] m.p. 150–153 °C (lit.: [18] 152–154 °C); $\alpha_{D}^{20}$ = +8.1° (c 0.25, CHCl_3_) [lit.: [18] $\alpha_{D}^{20}$ = +8.4° (c 0.33, CHCl_3_); MS (ESI, MeOH): m/z 541.2 (100%, [M + H]^+^).
*(3β)-28-[(2-Aminoethyl)amino]-20,28-dioxo-30-norlupan-3-yl acetate* (**20**). This compound (0.80 g, 88%) was obtained from **4** following GPB as a colorless solid; [42] m.p. 231–234 °C (lit.: [19] 230–234 °C); $\alpha_{D}^{20}$ = −8.5° (c 0.20, CHCl_3_) [lit.: [19] $\alpha_{D}^{20}$ = −8.5° (c 0.16, CHCl_3_); MS (ESI, MeOH): m/z 543.1 (100%, [M + H]^+^).
*(3β)-28-Oxo-piperazin-1-yl-olean-12-en-3-yl acetate* (**21**). This compound (0.91 g, 92%) was obtained from **1** following GPB as a colorless solid; [43,44,45,46] m.p. 170–175 °C (lit.: [24] 170–176 °C); MS (ESI, MeOH): m/z 567.4 (50%, [M + H]^+^).
*(3β)-28-Oxo-piperazin-1-yl-ursan-12-en-3-yl acetate* (**22**). This compound (0.85 g, 85%) was obtained from **2** following GPB as a colorless solid; [47,48] m.p. 157–160 °C (lit.: [24] 158–161 °C); MS (ESI, MeOH): m/z 567.3 (60%, [M + H]^+^).
*(3β)-28-Oxo-piperazin-1-yl-lup-20(29)-en-3-yl acetate* (**23**). This compound (0.90 g, 93%) was obtained from **3** following GPB as a colorless solid; [47,48] m.p. 177–180 °C (lit.: [24] 177–181 °C); MS (ESI, MeOH): m/z 567.3 (38%, [M + H]^+^).
*(3β)-20,28-Dioxo-piperazin-1-yl-30-norlupan-3-yl acetate* (**24**). This compound (0.82 g, 86%) was obtained from **4** following GPB as a colorless solid; [47,48] m.p. 115–123 °C (lit.: [24] 115–125 °C); MS (ESI, MeOH): m/z 569.2 (25%, [M + H]^+^).
*(3β)28-(1,4-Diazabicyclo[3.2.2]nonyl-4-yl)-28-oxoolean-12-en-3-yl acetate* (**25**). Following GPB from **1** (626 mg, 1.26 mmol) and **7** (500 mg, 2.51 mmol), **25** (462 mg, 73%) was obtained as colorless solid; m.p. 271–274 °C; R_f_ = 0.7 (CHCl_3_/MeOH, 9:1); [α]_D_ = +20.5° (c 0.15, CHCl_3_); IR (ATR): ν = 2943 br, 1732 m, 1621 m, 1463 w, 1393 m, 1363 m, 1243 s, 1174 m, 1140 m, 1115 w, 1026 m, 1005 m, 749 m cm^−1^; ^1^H NMR (400 MHz, CDCl_3_): δ = 6.15–5.99 (m, 2H, 34-H), 5.27–5.24 (m, 1H, 12-H), 4.76 (m, 1H, 39-H), 4.51–4.44 (m, 1H, 3-H), 4.38–3.99 (m, 5H, 35-H_a_ + 37-H_2_ + 40-H_2_), 3.75 (t, J = 12.0 Hz, 1H, 35-H_b_), 3.04 (d, J = 13.6 Hz, 1H, 18-H), 2.38–2.25 (m, 2H, 38-H_2_), 2.24–2.11 (m, 2H, 41-H_2_), 2.03 (s, 3H, 32-H_3_), 2.00–1.81 (m, 3H, 11-H_2_ + 16-H_a_), 1.61 (m, 7H, 1-H_a_ + 6-H_a_ + 9-H + 15-H_a_ + 19-H_a_ + 22-H_2_), 1.48–1.16 (m, 10H, 1-H_b_ + 2-H_2_ + 6-H_b_ + 7-H_2_ + 16-H_b_ + 19-H_b_ + 21-H_2_), 1.13 (s, 3H, 26-H_3_), 0.92 (s, 3H, 23-H_3_), 0.91 (s, 3H, 25-H_3_), 0.90 (s, 3H, 30-H_3_), 0.86 (s, 3H, 29-H_3_), 0.84 (s, 3H, 24H_3_), 0.81 (s, 1H, 5-H), 0.67 (s, 3H, 27-H_3_) ppm; ^13^C NMR (101 MHz, CDCl_3_): δ = 174.9 (C-28), 171.0 (C-31), 144.1 (C-13), 122.0 (C-12), 80.9 (C-3), 72.4 (C-34), 55.4 (C-35), 55.3 (C-5), 47.9 (C-37), 47.6 (C-10), 46.1 (C-17), 45.2 (C-39), 43.7 (C-18), 41.9 (C-14), 40.9 (C-40), 39.1 (C8), 38.1 (C-22), 37.7 (C-20), 37.0 (C-4), 33.8 (C-1), 33.5 (C-21), 32.9 (C-23), 32.8 (C-16), 32.5 (C-7), 28.0 (C-29), 27.9 (C-15), 25.8 (C-26), 24.0 (C-38), 23.5 (C-41), 23.3 (C-19), 22.6 (C-2), 22.5 (C-11), 21.3 (C-32), 18.2 (C-6), 17.0 (C-25), 16.6 (C-24), 15.4 (C-27), 14.1 (C-30), 8.6 (C-9) ppm; MS (ESI, MeOH): m/z = 607.5 (100%, [M + H]^+^), 608.5 (40%, [M + 2H]^+^); analysis calcd for C_39_H_62_N_2_O_3_ (606.94): C 77.18, H 10.30, N 4.62; found: C 76.84, H 10.58, N 4.45.
*(3β)28-(1,4-Diazabicyclo[3.2.2]non-4-yl)-28-oxolup-20(29)en-3-yl acetate* (**26**). Following GPB from **3** (250 mg, 0.50 mmol) and **7** (249 mg, 1.25 mmol), **26** (228 mg, 74%) was obtained as a colorless solid; m.p. 242–246 °C; R_f_ = 0.4 (DCM/MeOH, 9:1); [α]_D_ = −0.9° (c 0.17, CHCl_3_); IR (ATR): ν = 2941 m, 1731 m, 1624 m, 1475 m, 1398 m, 1385 m, 1242 s, 1117 m, 1028 m, 978 m, 749 m cm^−1^; ^1^H NMR (500 MHz, CDCl_3_): δ = 4.72 (d, J = 2.3 Hz, 1H, 29-H_a_), 4.62 (dq, J = 4.6, 2.5 Hz, 1H, 39-H), 4.58 (dt, J = 2.4, 1.4 Hz, 1H, 29-H_b_), 4.49–4.45 (m, 1H, 3-H), 3.79–3.64 (m, 2H, 34-H_2_), 3.16–2.88 (m, 7H, 13-H + 19-H_2_ + 35-H + 37-H_2_ + 40-H_2_), 2.12 (dt, J = 13.5, 3.5 Hz, 1H, 41-H_a_), 2.04 (s, 3H. 32-H_3_), 2.02–1.89 (m, 5H, 1-H_a_ + 16-H_a_ + 21-H_a_ + 22-H_a_ + 41-H_b_), 1.89–1.45 (m, 14H, 2-H_2_ + 7-H_2_ + 12-H_2_ + 15-H_a_ + 18-H_2_ + 30-H_3_ + 38-H_2_), 1.44–1.06 (m, 7H, 6-H_2_ + 9-H + 11-H_2_ + 16-H_b_ + 22-H_b_), 0.96 (s, 3H, 27-H_3_), 0.94 (s, 3H, 25-H_3_), 0.92–0.89 (m, 3H, 1-H_b_ + 15-H_b_ + 21-H_b_), 0.86 (s, 3H, 23-H_3_), 0.85 (s, 3H, 26-H_3_), 0.84 (s, 3H, 24-H_3_), 0.79 (dd, J = 8.7, 3.3 Hz, 1H, 5-H) ppm; ^13^C NMR (126 MHz, CDCl_3_): δ = 173.3 (C-28), 171.0 (C-31), 151.4 (C-20), 109.1 (C-29), 81.0 (C-3), 55.6 (C-5), 55.2 (C-17), 53.0 (C-18), 50.8 (C-9), 47.4 (C-39), 46.8 (C-37), 46.3 (C-35), 45.7 (C-19), 45.2 (C-34), 41.9 (C-14), 40.7 (C-8), 38.4 (C-1), 37.8 (C-4), 37.2 (C-10), 36.9 (C-13), 36.2 (C-22), 34.3 (C-7), 32.5 (C-40), 31.5 (C-16), 29.9 (C-21), 27.9 (C-23), 26.6 (C-15), 26.5 (C41 + C38), 25.6 (C-12), 23.7 (C-2), 21.3 (C-32), 21.2 (C-11), 19.7 (C-30), 18.2 (C-6), 16.5 (C-24), 16.3 (C-25), 16.1 (C-26), 14.7 (C-27) ppm; MS (ESI, MeOH): m/z = 607.6 (100%, [M + H]^+^), 608.6 (45%, [M + 2H]^+^), 1214.2 (5%, [2M + 2H]^+^); analysis calcd for C_39_H_62_N_2_O_3_ (606.94): C 77.18, H 10.30, N 4.62; found: C 76.97, H 10.51, N 4.44.
*(3β)28-(1,4-Diazabicyclo[3.2.2]non-4-yl)-20,28-dioxo-30-norlupan-3-yl acetate* (**27**). Following GPB from **4** (250 mg, 0.49 mmol) and **7** (238 mg, 1.19 mmol), **27** (270 mg, 99%) was obtained as a colorless solid; m.p. 253 °C (decomp.); R_f_ = 0.3 (DCM/MeOH/, 9:1); [α]_D_ = −8.7° (c 0.21, CHCl_3_); IR (ATR): ν = 2940 br, 1731 m, 1622 m, 1367 m, 1243 s, 1026 m, 978 m, 772 s cm^−1^; ^1^H NMR (400 MHz, CDCl_3_): δ = 4.60 (s, 1H, 39-H), 4.46 (dd, J = 10.5, 5.5 Hz, 1H, 3-H), 3.71 (m, 2H, 34-H_2_), 3.25 (td, J = 11.3, 3.5 Hz, 1H, 19-H), 3.18–2.88 (m, 6H, 35-H_2_ + 37-H_2_ + 40-H_2_), 2.79 (td, J = 12.0, 3.8 Hz, 1H, 13-H), 2.16 (s, 3H, 29-H_3_), 2.15–2.05 (m, 2H, 18-H + 21-H_a_), 2.03 (s, 3H, 32-H_3_), 2.01–1.80 (m, 2H, 16-H_a_ + 21-H_b_), 1.79–1.55 (m, 6H, 15-H_a_ + 16-H_b_ + 38-H_2_ + 41-H_2_), 1.54–1.11 (m, 14H, 1-H_a_ + 2-H_a_ + 6-H_2_ + 7-H_2_ + 9-H + 11-H_2_ + 12-H_2_ + 15-H_b_ + 22-H_2_), 1.05 (dd, J = 13.4, 3.5 Hz, 1H, 2-H_b_), 0.98 (s, 3H, 27-H_3_), 0.96 (s, 1H, 1-H_b_), 0.93 (s, 3H, 26-H_3_), 0.83 (m, 6H, 23-H_3_ + 25-H_3_), 0.82 (s, 3H, 24-H_3_), 0.81–0.76 (m, 1H, 5-H) ppm; ^13^C NMR (126 MHz CDCl_3_): δ = 213.1 (C-20), 173.3 (C-28), 171.0 (C-31), 80.9 (C-3), 55.5 (C-5), 55.1 (C-17), 52.9 (C-18), 50.7 (C-9), 50.1 (C-19), 47.4 (C-39), 46.8 (C-35), 46.2 (C-37), 46.2 (C-40), 44.9 (C-34), 41.8 (C-14), 40.6 (C-8), 38.4 (C-1), 37.8 (C-4), 37.1 (C-10), 37.1 (C-22), 35.9 (C-13), 35.7 (C-16), 34.2 (C-7), 32.0 (C-38), 30.3 (C-29), 29.9 (C-15), 28.9 (C-21), 27.9 (C-24), 27.5 (C-41), 27.3 (C-12), 23.7 (C-2), 21.3 (C-32), 21.2 (C-11), 18.1 (C-6), 16.5 (C-23), 16.2 (C-25), 16.0 (C-26), 14.7 (C-27) ppm; MS (ESI, MeOH): m/z = 609.5 (25%, [M + H]^+^); analysis calcd for C_38_H_60_N_2_O_4_ (608.91): C 74.96, H 9.93, N 4.60; found: C 74.72, H 10.13, N 4.48.
*(3β)28-(1,3-Diazabicyclo[3.2.2]nonyl-3-yl)-28-oxoolean-12-en-3-yl acetate* (**28**). Following GPB from **1** (375 mg, 0.75 mmol) and **8** (300 mg, 1.52 mmol), **28** (462 mg, 73%) was obtained as an off-white solid; m.p. 130 °C (decomp.); R_f_ = 0.3 (CHCl_3_/MeOH, 98:2); [α]_D_ = +10.5° (c 0.16, CHCl_3_); IR (ATR): ν = 3221 brw, 2942 brm, 1731 m, 1610 m, 1530 m, 1446 s, 1366 m, 1244 s, 1026 s, 655 s cm^−1^; ^1^H NMR (400 MHz, MeOH-d_4_): δ = 5.83 (ddt, J = 9.9, 3.7, 1.7 Hz, 1H, 34-H_a_), 5.72–5.66 (m, 1H, 34-H_b_), 5.36 (t, J = 3.7 Hz, 1H, 12-H), 4.45 (dd, J = 10.9, 5.0 Hz, 1H, 3-H), 3.46 (d, J = 6.5 Hz, 1H, 39-H_a_), 3.27 (d, J = 6.7 Hz, 1H, 39-H_b_), 3.21 (dt, J = 8.9, 2.8 Hz, 1H, 18-H), 2.84 (dq, J = 15.0, 5.9 Hz, 2H, 36-H_2_), 2.73 (m, 3H, 18-H + 40-H_2_), 2.28 (q, J = 2.5 Hz, 2H, 37-H_2_), 2.14–2.05 (m, 2H, 9-H + 11-H_a_), 2.02 (s, 3H, 32-H_3_), 1.97–1.87 (m, 4H, 2-H_2_ + 11-H_b_ + 15-H_a_) 1.84–1.73 (m, 2H, 6-H_a_ + 19-H_a_), 1.69–1.51 (m, 7H, 16-H_a_ + 21-H_2_ + 38-H_2_ + 41-H_2_), 1.51–1.20 (m, 7H, 1-H_2_ + 6-H_b_ + 7-H_2_ + 22-H_2_), 1.18 (s, 3H, 26-H_3_), 1.16–0.99 (m, 3H, 15-H_b_ + 16-H_b_ + 19-H_b_), 0.97 (s, 3H, 25-H_3_), 0.94 (s, 3H, 30-H_3_), 0.91 (s, 3H, 23-H_3_), 0.88 (s, 3H, 29-H_3_), 0.87 (s, 3H, 24-H_3_), 0.85 (s, 1H, 5-H), 0.79 (s, 3H, 27-H_3_) ppm; ^13^C NMR (101 MHz, MeOH-d_4_): δ = 179.3 (C-28), 171.5 (C-31), 143.7 (C-13), 124.9 (C-34), 123.2 (C-34),122.7 (C-12), 81.1 (C-3), 56.1 (C-40), 55.3 (C-5), 49.6 (C36), 47.1 (C-38), 46.3 (C-17), 46.2 (C-19), 41.5 (C-14), 41.3 (C-18), 39.3 (C-8), 37.9 (C-1), 37.3 (C-20), 36.7 (C-4), 35.7 (C-39), 34.1 (C-21), 33.7 (C-10), 32.8 (C-16), 32.5 (C-22), 32.1(C-23), 30.2(C-7), 27.2 (C-29), 27.1 (C-15), 25.0 (C-26), 24.6 (C-41),23.4 (C-38) 23.2 (C-37), 23.1 (C-11), 22.6 (C-30), 22.2 (C-2), 19.7 (C-32), 17.9 (C-6), 16.5 (C-27), 15.7 (C-24), 14.5 (C-25) ppm; MS (ESI, MeOH): m/z = 607.4 (100%, [M + H]^+^), 608.4 (60%, [M + 2H]^+^); analysis calcd for C_39_H_62_N_2_O_3_ (606.94): C 77.18, H 10.30, N 4.62; found: C 76.93, H 10.56, N 4.49.
*(3β)28-(1,3-Diazabicyclo[3.2.2]nonyl)-3-yl)-28-oxours-12-en-3-yl acetate* (**29**). Following GPB from **2** (375 mg, 0.75 mmol) and **8** (404 mg, 2.03 mmol), **29** (332 mg, 73%) was obtained as a colorless solid; m.p. 135–139 °C (decomp.); R_f_ = 0.35 (DCM/MeOH, 96:4); [α]_D_ = +28.6° (c 0.14, CHCl_3_); IR (ATR): ν = 2924 br, 1733 s, 1651 m, 1455 m, 1369 m, 1243 s, 1141 w, 1026 m, 653 m cm^−1^; ^1^H NMR (500 MHz, CDCl_3_): δ = 5.77 (m, 1H, 34-H_a_), 5.67 (m, 1H, 34-H_b_), 5.27 (t, J = 3.7 Hz, 1H, 12-H), 4.60–4.44 (dd, J = 10.0, 6.0 Hz, 1H, 3-H), 3.41–3.19 (m, 2H, 39-H_2_), 3.07 –2.82 (m, 2H, 36-H_2_), 2.64 (dt, J = 10.8, 5.2 Hz, 1H, 40-H_a_), 2.53 (dt, J = 9.0, 5.5 Hz, 2H, 18-H, 40-H_b_), 2.29–2.10 (m, 3H, 11-H_2_ + 16-H_a_), 2.06 (s, 3H, 32-H_3_), 2.02–1.83 (m, 6H, 19-H + 1-H_a_ + 2-H_2_ + 7-H_2_), 1.80–1.70 (m, 3H, 15-H_a_ + 37-H_2_), 1.64 (m, 4H, 6-H_a_ + 21-H_a_ + 22-H_2_), 1.59–1.25 (m, 10H, 6-H_b_ + 9-H + 16-H_b_ + 20-H_2_ + 21-H_b_ + 38-H_2_ + 41-H_2_), 1.09 (s, 3H, 27-H_3_), 1.08–0.95 (m, 2H, 1-H_b_ + 15-H_b_), 0.96 (s, 3H, 25-H_3_), 0.94 (s, 3H, 30-H_3_) 0.89 (s, 3H, 29-H_3_), 0.88 (s, 3H, 24-H_3_), 0.87 (s, 3H, 23-H_3_), 0.85–0.81 (m, 1H, 5-H), 0.79 (s, 3H, 26-H_3_) ppm; ^13^C NMR (126 MHz, CDCl_3_): δ = 178.1 (C-28), 171.0 (C-31), 139.2 (C-13), 125.8(C-12), 125.2 (C-34), 125.1 (C-34), 80.9 (C-3), 56.7 (C-18), 55.2(C-5), 53.9 (C-19), 52.3 (C-36), 50.3 (C-40), 47.8 (C-17), 47.5 (C-9), 42.4 (C-14), 39.8 (C-38), 39.6 (C-8), 39.1 (C-20), 38.3 (C-1), 37.7 (C-4), 37.3 (C-37), 36.9 (C-10), 36.2 (C-7), 36.2 (C-39), 32.7(C-22), 31.0 (C-21), 28.1 (C-24), 27.9 (C-16), 26.3 (C-11), 24.9 (C-41), 23.5 (C-2), 23.5 (C-15), 23.2 (C-27), 21.3 (C-32), 21.2 (C-25), 18.2 (C-6), 17.3 (C-29), 17.0 (C-26), 16.7 (C-23), 15.6 (C-30) ppm; MS (ESI, MeOH): m/z = 607.3 (100%, [M + H]^+^), 608.3 (65%; [M + 2H]^+^; m/z = 605.3 (100%, [M − H]^−^); analysis calcd for C_39_H_62_N_2_O_3_ (606.94): C 77.18, H 10.30, N 4.62; found: C 76.87, H 10.57, N 4.43.
*(3β)28-(1,3-Diazabicyclo[3.2.2]non-3-yl)-28-oxolup-20(29)-en-3-yl acetate* (**30**). Following GPB from **3** (200 mg, 0.40 mmol) and **8** (238 mg, 1.19 mmol), **30** (125 mg, 95%) was obtained as an amorphous colorless solid; R_f_ = 0.30 (DCM/MeOH, 98:2); [α]_D_ = +5.5° (c 0.17, CHCl_3_); IR (ATR): ν = 2942 m, 1732 m, 1638 m, 1450 m, 1368 m, 1243 s, 1027 m, 978 m, 881 m, 772 m, 653 m cm^−1^; ^1^H NMR (400 MHz, CDCl_3_): δ = 5.75 (m, 1H, 34-H_a_), 5.66 (m, 1H, 34-H_b_), 4.72 (d, J = 2.4 Hz, 1H, 29-H_a_), 4.59 (dt, J = 2.5, 1.4 Hz, 1H, 29-H_b_), 4.51–4.42 (m, 1H, 3-H), 3.37 (qt, J = 13.9, 6.8 Hz, 2H, 39-H_2_), 3.11–2.92 (m, 3H, 19-H + 36-H_a_ + 40-H_a_), 2.65–2.52 (m, 4H, 36-H_b_ + 40-H_b_), 2.37 (td, J = 12.4, 3.6 Hz, 1H, 9-H), 2.17 (tp, J = 5.7, 2.9, 2.3 Hz, 2H, 13-H + 18-H), 2.03 (s, 3H, 32-H_3_), 2.00–1.72 (m, 4H, 1-H_a_ + 12-H_a_ + 21-H_a_ + 22-H_a_), 1.68 (m, 4H, 21-H_b_ + 30-H_3_), 1.66–1.56 (m, 6H, 2-H_2_ + 6-H_a_ + 11-H_a_ + 15-H_a_ + 22-H_b_), 1.55–0.97 (m, 14H, 1-H_b_ + 6-H_b_ + 7-H_2_ + 11-H_b_ + 12-H_b_ + 15-H_b_ + 16-H_2_ + 37-H_2_ + 38-H + 41-H_2_), 0.95 (s, 3H, 27-H_3_), 0.93 (s, 3H, 26-H_3_), 0.84 (s, 3H, 23-H_3_), 0.83 (s, 3H, 25-H_3_), 0.82 (s, 3H, 24-H_3_), 0.80–0.74 (m, 1H, 5-H) ppm; ^13^C NMR (126 MHz, CDCl_3_): δ = 177.7 (C-28), 171.0 (C-31), 151.0 (C-20), 118.9 (C-34), 109.4 (C-29), 80.9 (C-3), 55.9 (C36), 55.4 (C-5), 50.5 (C-9), 50.3(C-40), 50.1 (C-18), 49.2 (C-17), 46.8 (C-19), 42.4 (C-14), 40.8 (C-8), 38.4 (C-13), 38.1 (C-1), 37.8 (C-4), 37.7 (C-38), 37.1 (C-10), 34.3 (C-22), 34.2 (C-7), 33.0 (C-16), 30.9 (C-21), 29.5 (C-15), 27.9 (C-23), 25.6 (C-12), 25.4 (C-41), 23.7 (C-2), 21.8 (C-37), 21.3 (C-32), 21.0 (C-11), 19.4 (C-30), 18.2 (C-6), 16.5 (C-24), 16.2 (C-25), 16.2 (C-26), 14.6 (C-27) ppm; MS (ESI, MeOH): m/z = 607.3 (100%, [M + H]^+^, 1241.9 (5%, [2M + 3H_2_O + 2H]^2+^); analysis calcd for C_39_H_62_N_2_O_3_ (606.94): C 77.18, H 10.30, N 4.62; found: C 76.81, H 10.53, N 4.41.
*(3β)28-(1,4-Diazabicyclo[3.2.2]non-4-yl)-20,28-dioxo-30-norlupan-3-yl-acetate* (**31**). Following GPB from **4** (250 mg, 0.39 mmol) and **8** (238 mg, 1.19 mmol), **31** (178 mg, 73%) was obtained as a colorless solid; m.p. 130–132 °C; R_f_ = 0.40 (DCM/MeOH, 95:5); [α]_D_ = −7.0° (c 0.15, CHCl_3_); IR (ATR): ν = 2942 brm, 1732 m, 1656 m, 1517 m, 1449 m, 1363 m, 1244 m, 1195 m, 1026 m, 979 m, 772 m, 654 m cm^−1^; ^1^H NMR (500 MHz, CDCl_3_): δ = 5.89–5.64 (m, 2H, 34-H_2_), 4.45 (dd, J = 11.0, 5.2 Hz, 1H, 3-H), 3.52 (m, 2H, 39-H_2_), 3.37 (m, 2H, 36-H_2_), 3.33–3.18 (m, 1H, 19-H), 2.87 (dt, J = 26.8, 6.1 Hz, 2H, 40-H_2_), 2.42–2.30 (m, 2H, 37-H_2_), 2.29–2.17 (m, 1H, 13-H), 2.14 (s, 3H, 29-H_3_), 2.12–2.04 (m, 2H, 18-H + 38-H), 2.02 (s, 3H, 32-H_3_), 2.00–1.81 (m, 1H, 21-H_a_), 1.67–1.11 (m, 18H, 1-H_a_ + 2-H_a_ + 6-H_2_ + 7-H_2_ + 9-H + 11-H_2_ + 12-H_2_ + 15-H_2_ + 16-H_a_ + 22-H_2_ + 41-H_2_), 1.05 (m, 3H, 2-H_b_ + 16-H_b_ + 21-H_b_), 0.97 (s, 3H, 27-H_3_), 0.94–0.91 (m, 1H, 1-H_b_), 0.89 (s, 3H, 26-H_3_), 0.84–0.80 (m, 9H, 23-H_3_ + 24-H_3_ + 25-H_3_), 0.79–0.73 (m, 1H, 5-H) ppm; ^13^C NMR (126 MHz, CDCl_3_): δ = 212.7 (C-20), 176.8 (C-28), 170.9 (C-31), 125.6 (C-34), 80.8 (C-3), 56.2 (C36), 55.7 (C-17), 55.4 (C-5), 51.3 (C-4), 51.2 (C-19), 50.4 (C-9), 50.0 (C-18), 49.4 (C-40), 42.2 (C-14), 40.7 (C-8), 38.3 (C-1), 37.8 (C-38), 37.8 (C-22), 37.1 (C-10), 36.8 (C-13), 35.0 (C-39), 34.2 (C-7), 32.6 (C-16), 30.1 (C-29), 29.5 (C-15), 28.6 (C-21), 27.9 (C-24), 27.9 (C-25), 27.7 (C-12), 27.2 (C-41), 23.8 (C-2), 23.6 (C-37), 21.3 (C-32), 20.9 (C-11), 18.2 (C-6), 16.5 (C-23), 16.2 (C-26), 14.6 (C-27) ppm; MS (ESI, MeOH): m/z = 609.2 (100%, [M + H]^+^), 610.2 (50%; [M + 2H]^+^); analysis calcd for C_38_H_60_N_2_O_4_ (608.91): C 74.96, H 9.93, N 4.60; found: C 74.76, H 10.14, N 4.41.

## Figures and Tables

**Table 1:** Cytotoxicity of parent compounds **OA**, **UA**, **BA**, **PA**, acetates **1**–**4**, starting materials **5**–**8** as well as products **9**–**16** (EC_50_-values in µM from SRB-assays) after 72 h of treatment, the values are averaged from three independent experiments performed each in triplicate, confidence interval CI = 95%; mean ± standard mean error); n.d. not determined; n.s. not soluble; betulinic acid (**BA**) and doxorubicin (**DX**) were used as positive controls. Cell lines: malignant: A375 (melanoma), HT29 (colon adenocarcinoma), MCF-7 (breast adenocarcinoma), A2780 (ovarian carcinoma), FaDu (hypopharyngeal carcinoma); nonmalignant: NIH 3T3 (fibroblasts).

Compound	A375	HT29	MCF-7	A2780	FaDu	NIH 3T3
**OA**	>30	>30	>30	>30	>30	>30
**UA**	15.4 ± 1.0	12.4 ± 1.1	14.7 ± 0.4	17.3 ± 0.9	18.2 ± 1.7	16.3 ± 1.4
**BA**	17.7 ± 0.4	16.8 ± 2.0	12.3 ± 1.1	9.4 ± 1.1	13.7 ± 0.9	19.3 ± 1.1
**PA**	>30	>30	>30	>30	>30	>30
**1**	13.1 ± 1.1	20.5 ± 1.7	12.9 ± 1.9	9.4 ± 0.5	11.8 ± 0.9	17.5 ± 1.5
**2**	11.4 ± 1.4	17.3 ± 1.5	12.1 ± 1.2	8.3 ± 0.9	10.7 ± 0.8	16.4 ± 1.7
**3**	19.2 ± 1.7	21.3 ± 2.0	11.0 ± 0.5	18.3 ± 0.5	7.2 ± 1.2	>30
**4**–**8**	>30	>30	>30	>30	>30	>30
**9**	1.8 ± 0.3	1.7 ± 0.2	2.0 ± 0.3	2.1 ± 0.2	2.0 ± 0.1	1.8 ± 0.4
**10**	2.4 ± 0.2	2.9 ± 0.1	2.8 ± 0.6	2.7 ± 0.2	2.9 ± 0.2	2.2 ± 0.2
**11**	1.9 ± 0.2	2.6 ± 0.1	2.5 ± 0.4	2.6 ± 0.3	2.6 ± 0.1	1.9 ± 0.3
**12**	1.9 ± 0.3	2.4 ± 0.1	2.4 ± 0.4	2.3 ± 0.2	2.5 ± 0.1	1.9 ± 0.2
**13**	0.9 ± 0.1	0.6 ± 0.1	1.3 ± 0.4	1.0 ± 0.1	1.0 ± 0.1	1.0 ± 0.1
**14**	1.3 ± 0.2	0.8 ± 0.1	1.2 ± 0.4	1.2 ± 0.35	1.1 ± 0.2	1.1 ± 0.1
**15**	0.9 ± 0.1	2.1 ± 0.1	2.8 ± 0.4	1.8 ± 0.2	1.5 ± 0.1	0.5 ± 0.1
**16**	n.s.	n.s.	n.s.	n.s.	n.s.	n.s.
**DX**	n.d.	0.9 ± 0.2	1.1 ± 0.3	0.02 ± 0.01	1.7 ± 0.3	0.06 ± 0.03

**Table 2:** (EC_50_ in μM from SRB) of ethylenediamine amides **17**–**20** and piperazine derived amides **21**–**24**. The values are averaged from three independent experiments performed each in triplicate, confidence interval CI = 95%; mean ± standard mean error).

Compound	HT29	MCF-7	A2780	NIH 3T3
**17**	2.0 ± 0.2	1.7 ± 0.2	3.1 ± 0.1	2.1 ± 0.1
**18**	1.8 ± 0.1	2.0 ± 0.1	2.3 ± 0.1	2.6 ± 0.3
**19**	1.0 ± 0.3	1.3 ± 0.1	1.4 ± 0.2	1.4 ± 0.1
**20**	3.3 ± 1.2	3.1 ± 0.1	3.2 ± 0.2	2.1 ± 0.1
**21**	1.3 ± 0.1	1.7 ± 0.2	1.7 ± 0.1	1.7 ± 0.1
**22**	1.9 ± 0.3	2.0 ± 0.1	2.1 ± 0.1	2.1 ± 0.1
**23**	1.0 ± 0.1	1.4 ± 0.1	1.9 ± 0.1	0.9 ± 0.1
**24**	2.4 ± 0.3	2.8 ± 0.1	3.1 ± 0.1	0.7 ± 0.1

**Table 3:** (EC_50_ in μM from SRB) of diazabicyclononanes derived amides **25**–**31**. The values are averaged from three independent experiments performed each in triplicate, confidence interval CI = 95%; mean ± standard mean error); n.s. not soluble.

Compound	A375	HT29	MCF-7	A2780	FaDu	NIH 3T3
**25**, **26**	n.s.	n.s.	n.s.	n.s.	n.s.	n.s.
**27**	2.3 ± 0.2	5.2 ± 0.2	4.2 ± 0.8	3.9 ± 0.4	2.7 ± 0.4	2.2 ± 0.2
**28**, **29**	n.s.	n.s.	n.s.	n.s.	n.s.	n.s.
**30**	4.7 ± 0.2	4.8 ± 0.3	6.0 ± 0.9	5.5 ± 0.4	6.3 ± 0.5	9.3 ± 0.7
**31**	6.0 ± 0.5	8.2 ± 0.3	6.3 ± 0.5	6.4 ± 0.3	6.2 ± 0.6	5.0 ± 0.4

## Data Availability

Not applicable.

## Supplementary Materials

The Supplementary Materials are available online: representative ^1^H-, ^13^C NMR as well as IR (ATR) spectra.

## Sample Availability

Samples of the compounds are available from the authors.

## References

[1] Al-Harrasi A., Khan A.L., Rehman N.U., Csuk R. (2021). Biosynthetic diversity in triterpene cyclization within the Boswellia genus. Phytochemistry.

[2] Almahli H. (2020). Pentacyclic triterpene acids: Use, mode of action, biological activity and synthesis. J. Chem. Biol. Phys. Sci..

[3] Ayeleso T.B., Matumba M.G., Mukwevho E. (2017). Oleanolic acid and its derivatives: Biological activities and therapeutic potential in chronic diseases. Molecules.

[4] Ghiulai R., Rosca O.J., Antal D.S., Mioc M., Mioc A., Racoviceanu R., Macasoi I., Olariu T., Dehelean C., Cretu O.M. (2020). Tetracyclic and pentacyclic triterpenes with high therapeutic efficiency in wound healing approaches. Molecules.

[5] Hordyjewska A., Ostapiuk A., Horecka A., Kurzepa J. (2019). Betulin and betulinic acid: Triterpenoids derivatives with a powerful biological potential. Phytochem. Rev..

[6] Khwaza V., Oyedeji O.O., Aderibigbe B.A. (2020). Ursolic acid-based derivatives as potential anti-cancer agents: An update. Int. J. Mol. Sci..

[7] Rios J.L., Manez S. (2018). New Pharmacological Opportunities for Betulinic Acid. Planta Med..

[8] Salvador J.A.R., Leal A.S., Valdeira A.S., Goncalves B.M.F., Alho D.P.S., Figueiredo S.A.C., Silvestre S.M., Mendes V.I.S. (2017). Oleanane-, ursane-, and quinone methide friedelane-type triterpenoid derivatives: Recent advances in cancer treatment. Eur. J. Med. Chem..

[9] Sharma N., Palia P., Chaudhary A., Shalini, Verma K., Kumar I. (2020). A review on pharmacological activities of lupeol and its triterpene derivatives. J. Drug Deliv. Ther..

[10] Sun Q., He M., Zhang M., Zeng S., Chen L., Zhou L., Xu H. (2020). Ursolic acid: A systematic review of its pharmacology, toxicity and rethink on its pharmacokinetics based on PK-PD model. Fitoterapia.

[11] Lowitz J.T. (1788). Über eine neue fast benzoesäureartige Substanz der Birken. Crell’s Ann. Chem..

[12] Pisha E., Chai H., Lee I.S., Chagwedera T.E., Farnsworth N.R., Cordell G.A., Beecher C.W.W., Fong H.H.S., Kinghorn A.D., Brown D.M. (1995). Discovery of Betulinic Acid as a Selective Inhibitor of Human-Melanoma That Functions by Induction of Apoptosis. Nat. Med..

[13] Feng J.-H., Chen W., Zhao Y., Ju X.-L. (2009). Anti-tumor activity of oleanolic, ursolic and glycyrrhetinic acid. Open Nat. Prod. J..

[14] Paduch R., Kandefer-Szerszen M. (2014). Antitumor and Antiviral Activity of Pentacyclic Triterpenes. Mini-Rev. Org. Chem..

[15] Paszel-Jaworska A., Romaniuk A., Rybczynska M. (2014). Molecular Mechanisms of Biological Activity of Oleanolic Acid—A Source of Inspiration for A New Drugs Design. Mini-Rev. Org. Chem..

[16] Csuk R. (2014). Betulinic acid and its derivatives: A patent review (2008–2013). Expert Opin. Ther. Pat..

[17] Hoenke S., Heise N.V., Kahnt M., Deigner H.-P., Csuk R. (2020). Betulinic acid derived amides are highly cytotoxic, apoptotic and selective. Eur. J. Med. Chem..

[18] Kahnt M., Fischer L., Al-Harrasi A., Csuk R. (2018). Ethylenediamine derived carboxamides of betulinic and ursolic acid as potential cytotoxic agents. Molecules.

[19] Kahnt M., Heller L., Al-Harrasi A., Schaefer R., Kluge R., Wagner C., Otgonbayar C., Csuk R. (2018). Platanic acid-derived methyl 20-amino-30-norlupan-28-oates are potent cytotoxic agents acting by apoptosis. Med. Chem. Res..

[20] Kahnt M., Heller L., Grabandt P., Al-Harrasi A., Csuk R. (2018). Platanic acid: A new scaffold for the synthesis of cytotoxic agents. Eur. J. Med. Chem..

[21] Siewert B., Pianowski E., Csuk R. (2013). Esters and amides of maslinic acid trigger apoptosis in human tumor cells and alter their mode of action with respect to the substitution pattern at C-28. Eur. J. Med. Chem..

[22] Siewert B., Pianowski E., Obernauer A., Csuk R. (2014). Towards cytotoxic and selective derivatives of maslinic acid. Bioorg. Med. Chem..

[23] Wolfram R.K., Fischer L., Kluge R., Stroehl D., Al-Harrasi A., Csuk R. (2018). Homopiperazine-rhodamine B adducts of triterpenoic acids are strong mitocans. Eur. J. Med. Chem..

[24] Sommerwerk S., Heller L., Kerzig C., Kramell A.E., Csuk R. (2017). Rhodamine B conjugates of triterpenoic acids are cytotoxic mitocans even at nanomolar concentrations. Eur. J. Med. Chem..

[25] Hoenke S., Serbian I., Deigner H.-P., Csuk R. (2020). Mitocanic Di- and triterpenoid rhodamine B conjugates. Molecules.

[26] Kahnt M., Loesche A., Serbian I., Hoenke S., Fischer L., Al-Harrasi A., Csuk R. (2019). The cytotoxicity of oleanane derived aminocarboxamides depends on their aminoalkyl substituents. Steroids.

[27] De Costa B.R., He X., Linders J.T.M., Dominguez C., Gu Z.Q., Williams W., Bowen W. (1993). Synthesis and evaluation of conformationally restricted N-[2-(3,4-dichlorophenyl)ethyl]-N-methyl-2-(1-pyrrolidinyl)ethylamines at sigma receptors. 2. Piperazines, bicyclic amines, bridged bicyclic amines, and miscellaneous compounds. J. Med. Chem..

[28] Mikhlina E.E., Rubtsov M.V. (1963). Reaction of 3-quinuclidone with hydrazoic acid. Zh. Obshch. Khim..

[29] Takemoto T., Kometani K. (1965). Triterpene glycosides (mubenins) from the seeds of Stauntonia hexaphylla. Justus Liebigs Ann. Chem..

[30] Ewen E.S., Spring F.S. (1943). Triterpene resinols and related acids. XIV. The oxidation of acetylursolic acid. J. Chem. Soc..

[31] Taketa A.T.C., Breitmaier E., Schenkel E.P. (2004). Triterpenes and triterpenoidal glycosides from the fruits of Ilex paraguariensis (Mate). J. Braz. Chem. Soc..

[32] Petrenko N.I., Elantseva N.V., Petukhova V.Z., Shakirov M.M., Shul’ts E.E., Tolstikov G.A. (2002). Synthesis of Betulonic Acid Derivatives Containing Amino-Acid Fragments. Chem. Nat. Compd..

[33] Thibeault D., Gauthier C., Legault J., Bouchard J., Dufour P., Pichette A. (2007). Synthesis and structure-activity relationship study of cytotoxic germanicane- and lupane-type 3β-O-monodesmosidic saponins starting from betulin. Bioorg. Med. Chem..

[34] Vystrcil A., Budesinsky M. (1970). Triterpenes. XVI. Unusual epimerization of the C-19 acetyl group in 20-oxo-30-norlupane derivatives. Collect. Czech. Chem. Commun..

[35] Brandes B., Hoenke S., Fischer L., Csuk R. (2020). Design, synthesis and cytotoxicity of BODIPY FL labelled triterpenoids. Eur. J. Med. Chem..

[36] Loesche A., Kahnt M., Serbian I., Brandt W., Csuk R. (2019). Triterpene-based carboxamides act as good inhibitors of butyrylcholinesterase. Molecules.

[37] Bai K.-K., Yu Z., Chen F.-L., Li F., Li W.-Y., Guo Y.-H. (2012). Synthesis and evaluation of ursolic acid derivatives as potent cytotoxic agents. Bioorg. Med. Chem. Lett..

[38] Becker C.S., Chukanov N.V., Grigor’ev I.A. (2015). New Amino-Bisphosphonate Building Blocks in the Synthesis of Bisphosphonic Derivatives Based on Lead Compounds. Phosphorus Sulfur Silicon Relat. Elem..

[39] Kahnt M., Hoenke S., Fischer L., Al-Harrasi A., Csuk R. (2019). Synthesis and cytotoxicity evaluation of DOTA-conjugates of ursolic acid. Molecules.

[40] Wang J., Jiang Z., Xiang L., Li Y., Ou M., Yang X., Shao J., Lu Y., Lin L., Chen J. (2014). Synergism of ursolic acid derivative US597 with 2-deoxy-D-glucose to preferentially induce tumor cell death by dual-targeting of apoptosis and glycolysis. Sci. Rep..

[41] Pettit G.R., Melody N. (2019). Betulastatin Compounds for Cancer Cell Growth Inhibition. International Patent Application No.

[42] Heller L., Kahnt M., Loesche A., Grabandt P., Schwarz S., Brandt W., Csuk R. (2017). Amino derivatives of platanic acid act as selective and potent inhibitors of butyrylcholinesterase. Eur. J. Med. Chem..

[43] Brandes B., Koch L., Hoenke S., Deigner H.-P., Csuk R. (2020). The presence of a cationic center is not alone decisive for the cytotoxicity of triterpene carboxylic acid amides. Steroids.

[44] Friedrich S., Serbian I., Hoenke S., Wolfram R.K., Csuk R. (2020). Synthesis and cytotoxic evaluation of malachite green derived oleanolic and ursolic acid piperazineamides. Med. Chem. Res..

[45] Kahnt M., Wiemann J., Fischer L., Sommerwerk S., Csuk R. (2018). Transformation of asiatic acid into a mitocanic, bimodal-acting rhodamine B conjugate of nanomolar cytotoxicity. Eur. J. Med. Chem..

[46] Nie W., Luo J.-G., Wang X.-B., Yin H., Sun H.-B., Yao H.-Q., Kong L.-Y. (2011). Synthesis of new α-glucosidase inhibitors based on oleanolic acid incorporating cinnamic amides. Chem. Pharm. Bull..

[47] Hua S.-X., Huang R.-Z., Ye M.-Y., Pan Y.-M., Yao G.-Y., Zhang Y., Wang H.-S. (2015). Design, synthesis and in vitro evaluation of novel ursolic acid derivatives as potential anticancer agents. Eur. J. Med. Chem..

[48] Yang X., Li Y., Jiang W., Ou M., Chen Y., Xu Y., Wu Q., Zheng Q., Wu F., Wang L. (2015). Synthesis and Biological Evaluation of Novel Ursolic acid Derivatives as Potential Anticancer Prodrugs. Chem. Biol. Drug Des..

