# Supplementary material for: The Presence of a Cyclohexyldiamine Moiety Confers Cytotoxicity to Pentacyclic Triterpenoids
Source: Molecules. 2021 Apr 6;26(7):2102. doi: 10.3390/molecules26072102 (PMC8038856; doi:10.3390/molecules26072102)

Supplementary Materials: The Presence of a Cyclohexyldiamine Moiety Confers Cytotoxicity to Pentacyclic Triterpenoids

Sophie Hoenke [1], Martin A. Christoph [1], Sander Friedrich [1], Niels Heise [1], Benjamin Brandes [1], Hans-Peter Deigner [2], Ahmed Al-Harrasi [3], and René Csuk [1,*]

1 Organic Chemistry, Martin–Luther University Halle–Wittenberg, Kurt–Mothes, Str. 2, D-06120 Halle (Saale), Germany; sophie.hoenke@chemie.uni-halle.de (S.H.); martin.christoph@student.uni-halle.de (M.A.C.); sander.friedrich@student.uni-halle.de (S.F.); niels.heise@student.uni-halle.de (N.H.); Benjamin.brandes@chemie.uni-halle.de (B.B.)

2 Institute of Precision Medicine, Medical and Life Science Faculty, Furtwangen University, Jakob–Kienzle–Str. 17, D-78054 Villigen–Schwenningen, Germany; dei@hs-furtwangen.de

3 Chair of Oman's Medicinal Plants and Marine Natural Products, University of Nizwa, P.O. Box 33, Birkat Al-Mauz, PC 616 Nizwa, Oman; aharrasi@unizwa.edu.om

* Correspondence: rene.csuk@chemie.uni-halle.de; Tel.: +49-345-5525660

NMR spectra (including numbering scheme) of selected compounds

Spectra of 9

^{1}H NMR (500 MHz, $CDCl_3$)

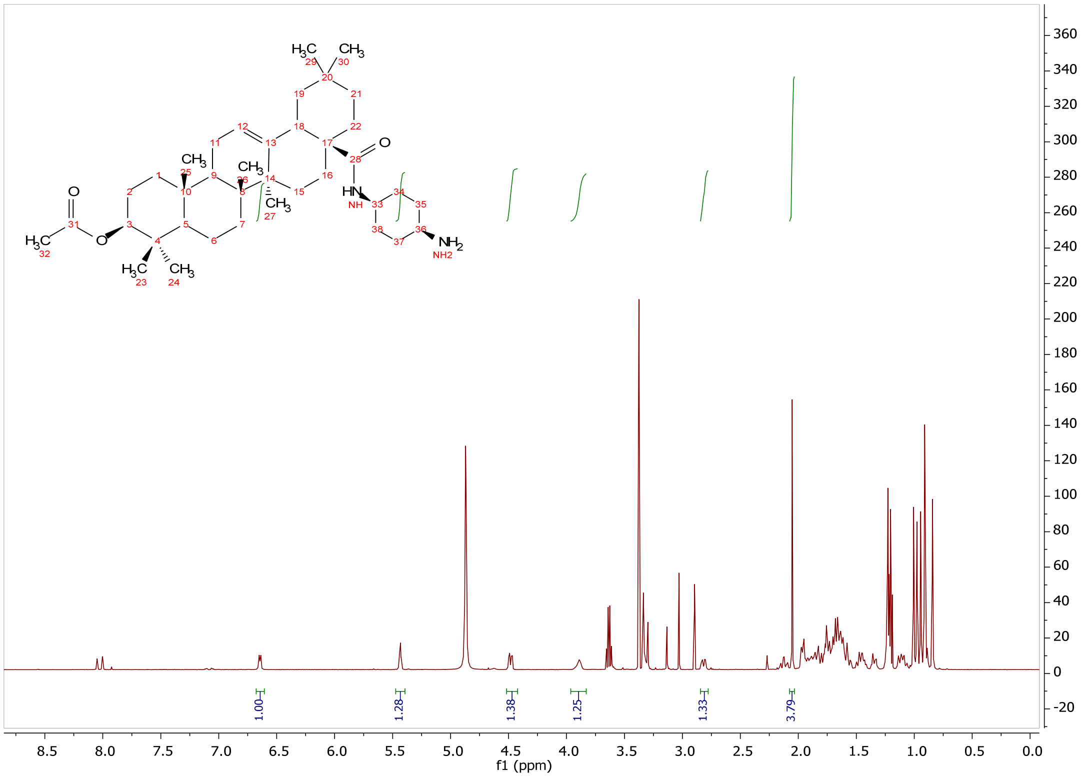

^{13}C APT-NMR (126 MHz, $CDCl_3$)

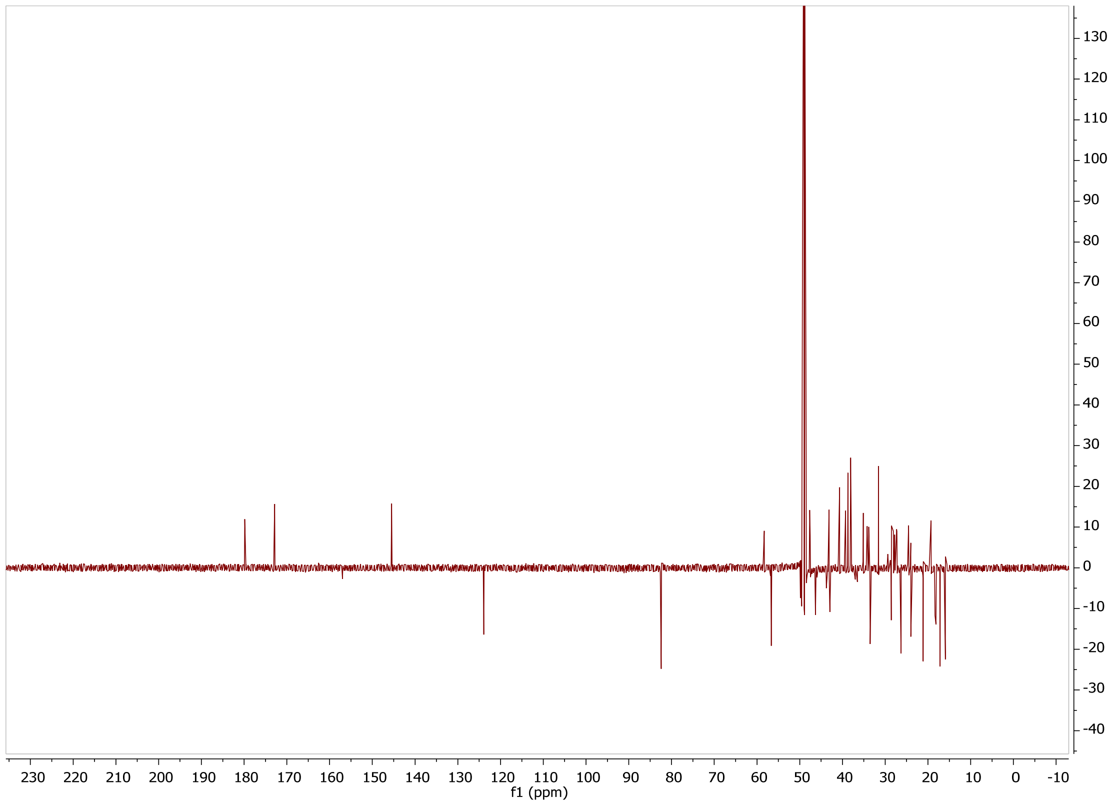

Spectra of 10

^{1}H NMR (400 MHz, $CDCl_3$)

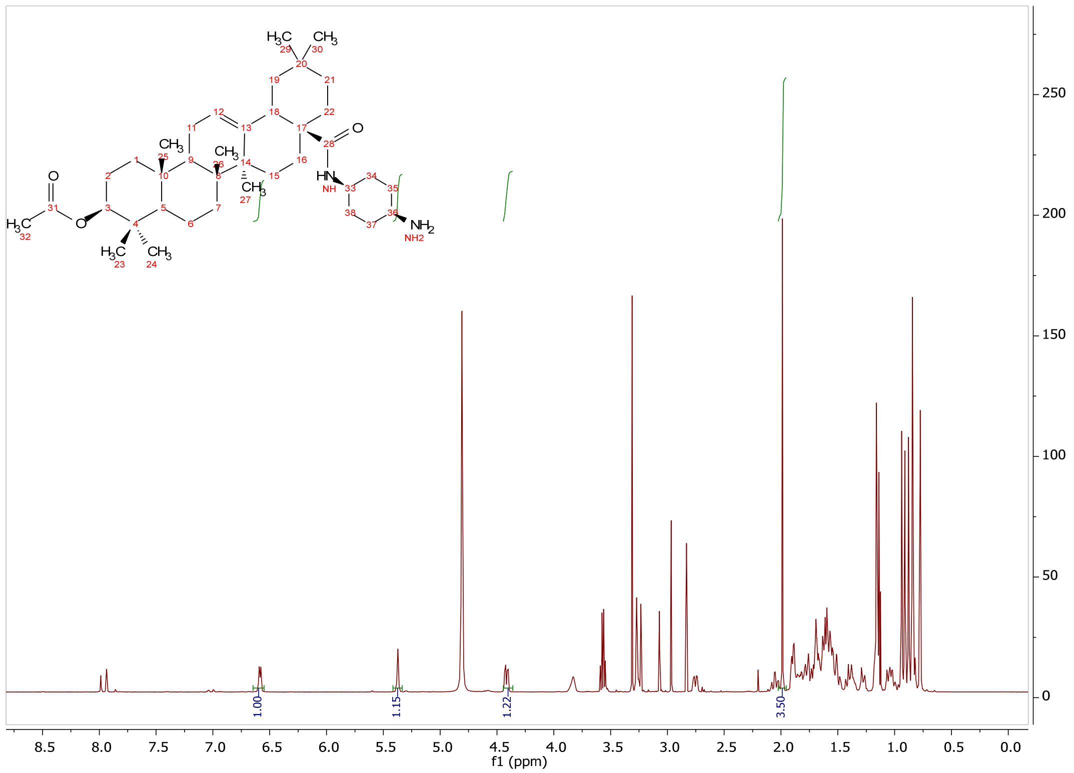

^{13}C APT-NMR (101 MHz, $CDCl_3$)

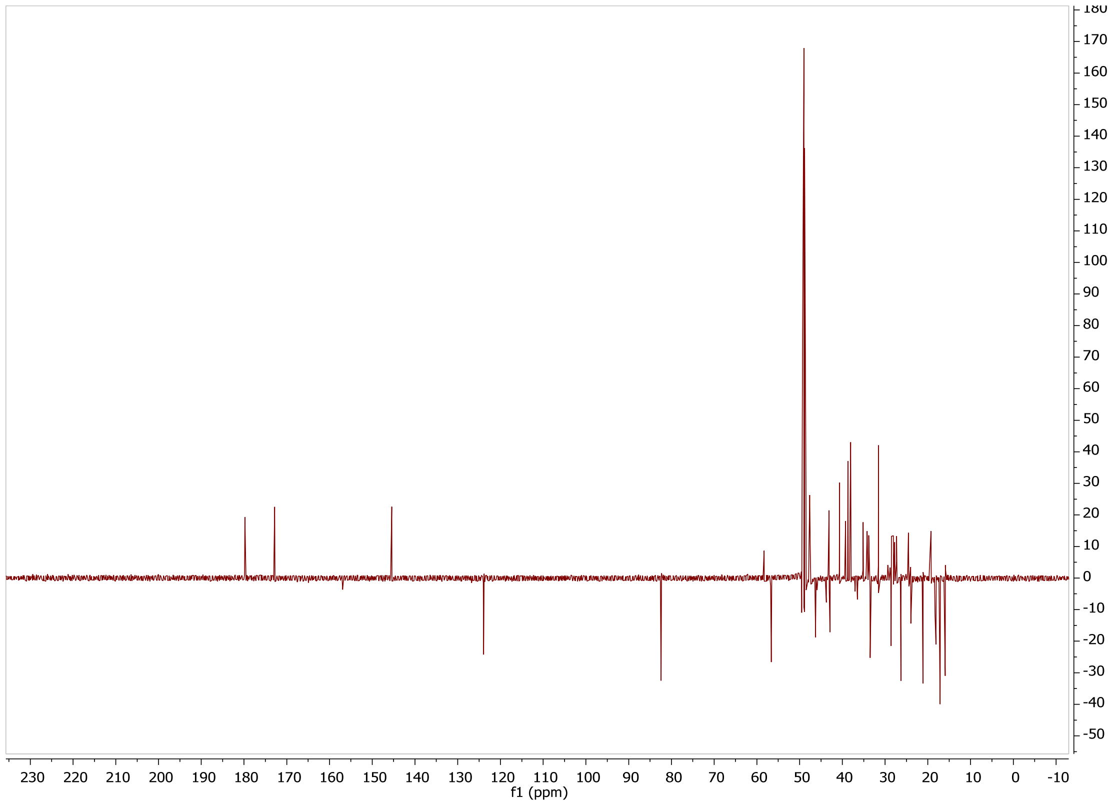

Spectra of 11

^{1}H NMR (500 MHz, $CDCl_3$)

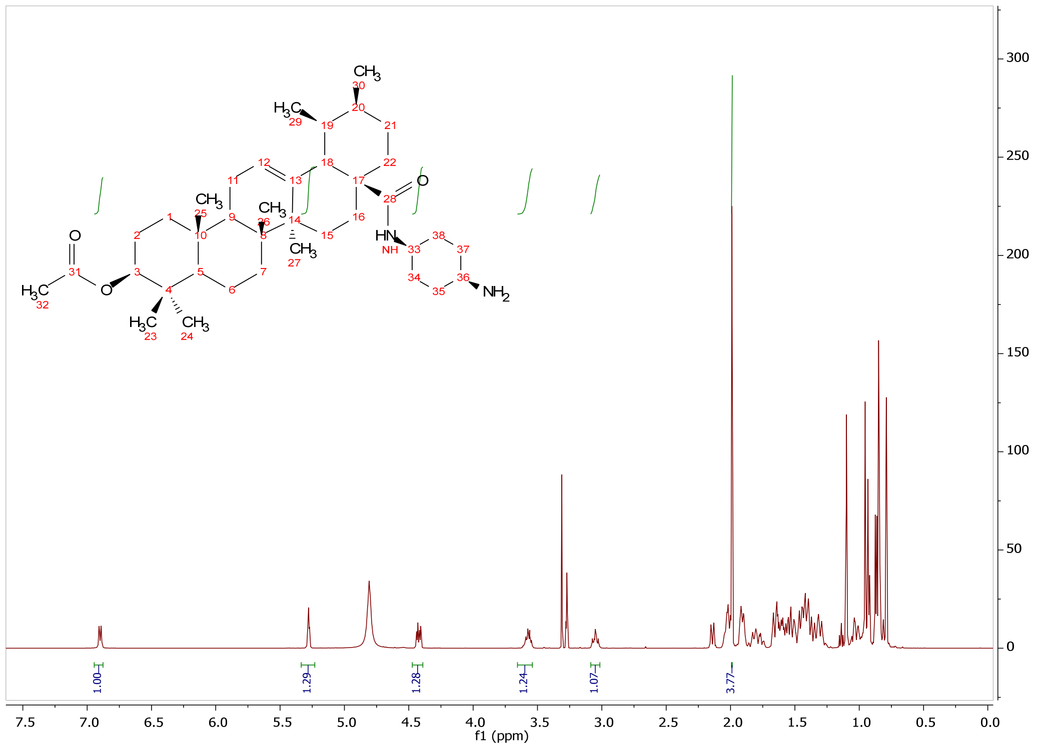

^{13}C APT-NMR (126 MHz, $CDCl_3$)

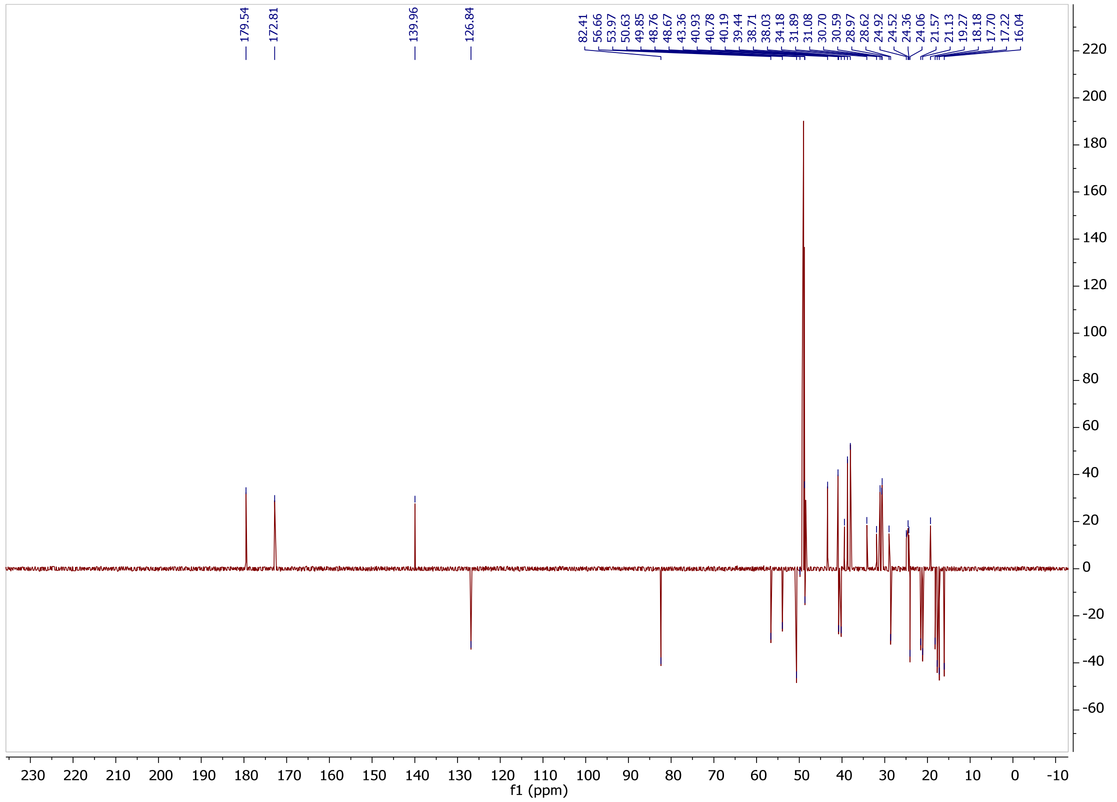

Spectra of 12

^{1}H NMR (500 MHz, $CDCl_3$)

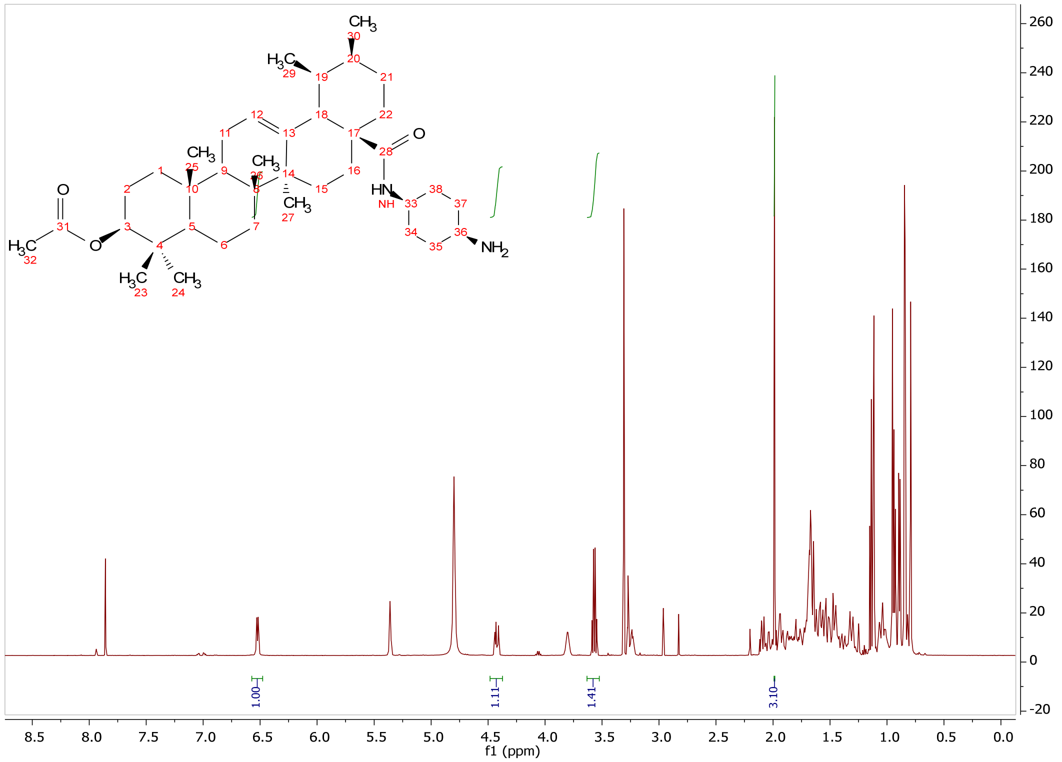

^{13}C APT-NMR (126 MHz, $CDCl_3$)

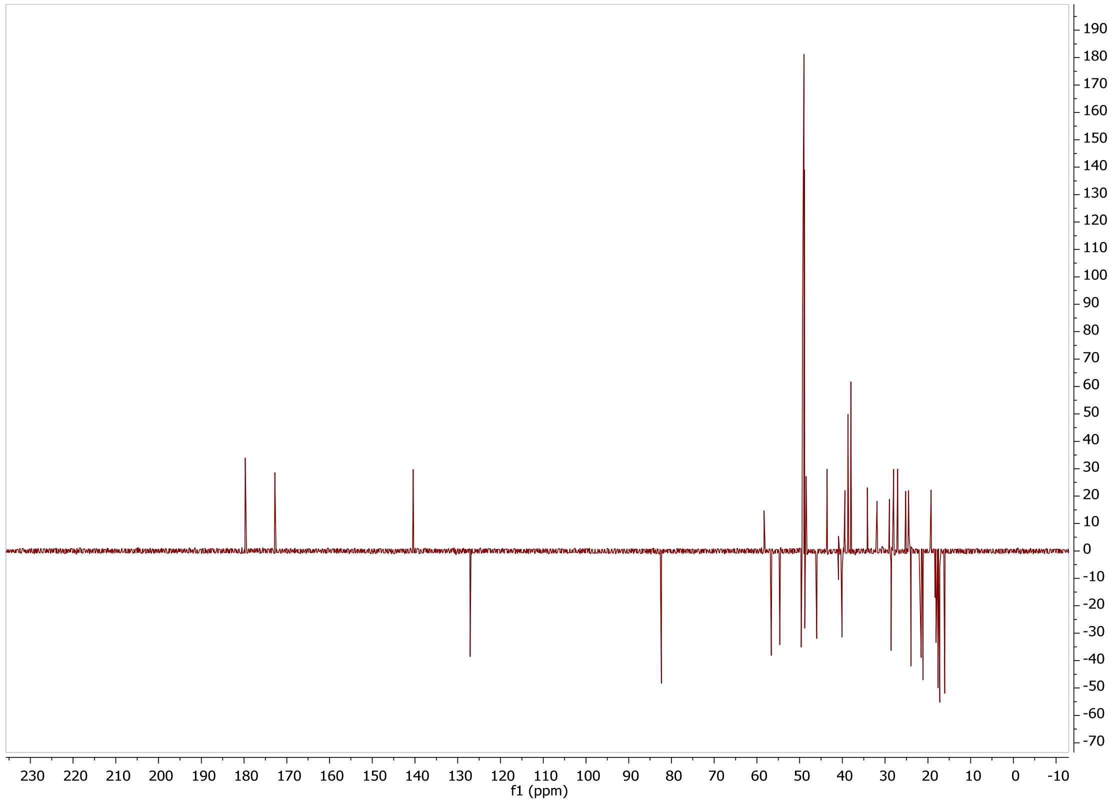

Spectra of 13

^{1}H NMR (400 MHz, $CDCl_3$)

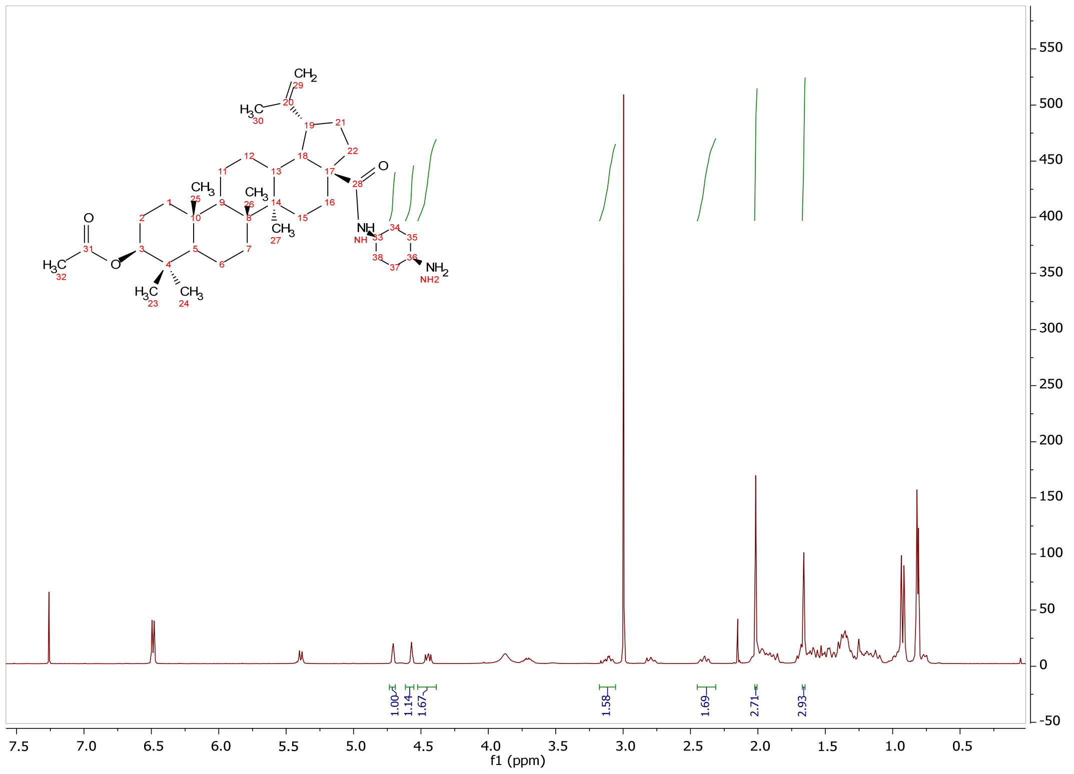

^{13}C APT-NMR (101 MHz, $CDCl_3$)

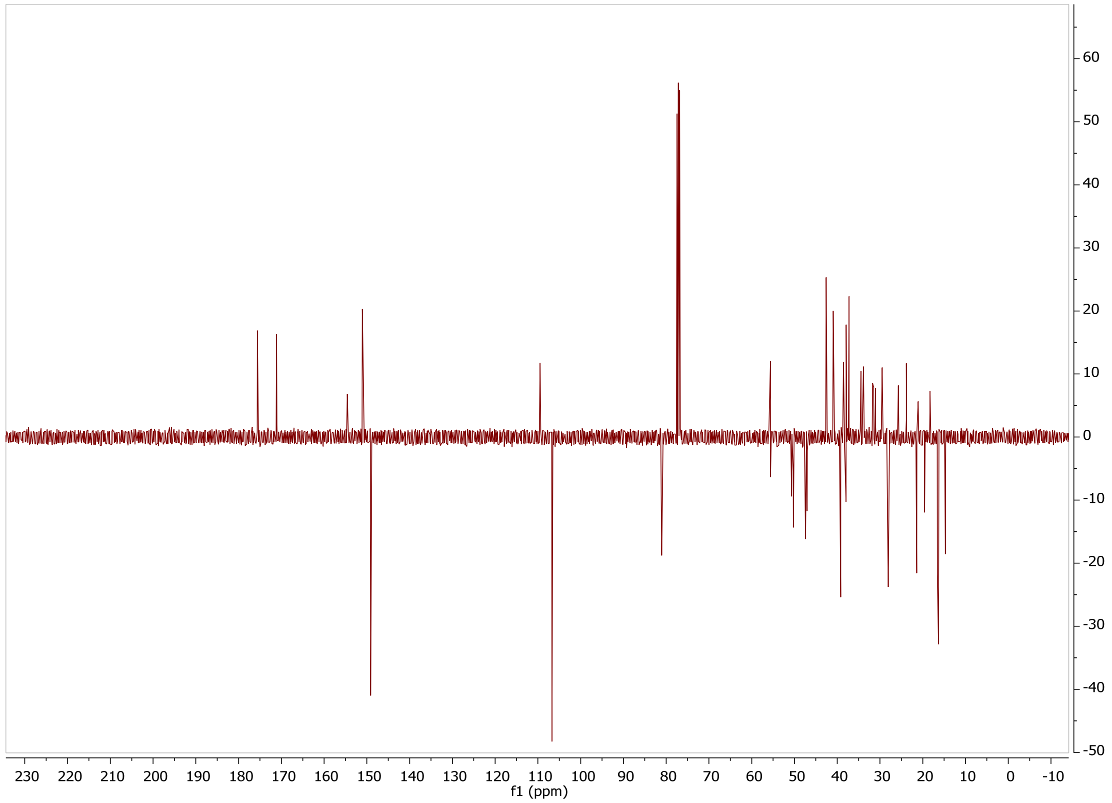

Spectra of 14

^{1}H NMR (400 MHz, $CDCl_3$)

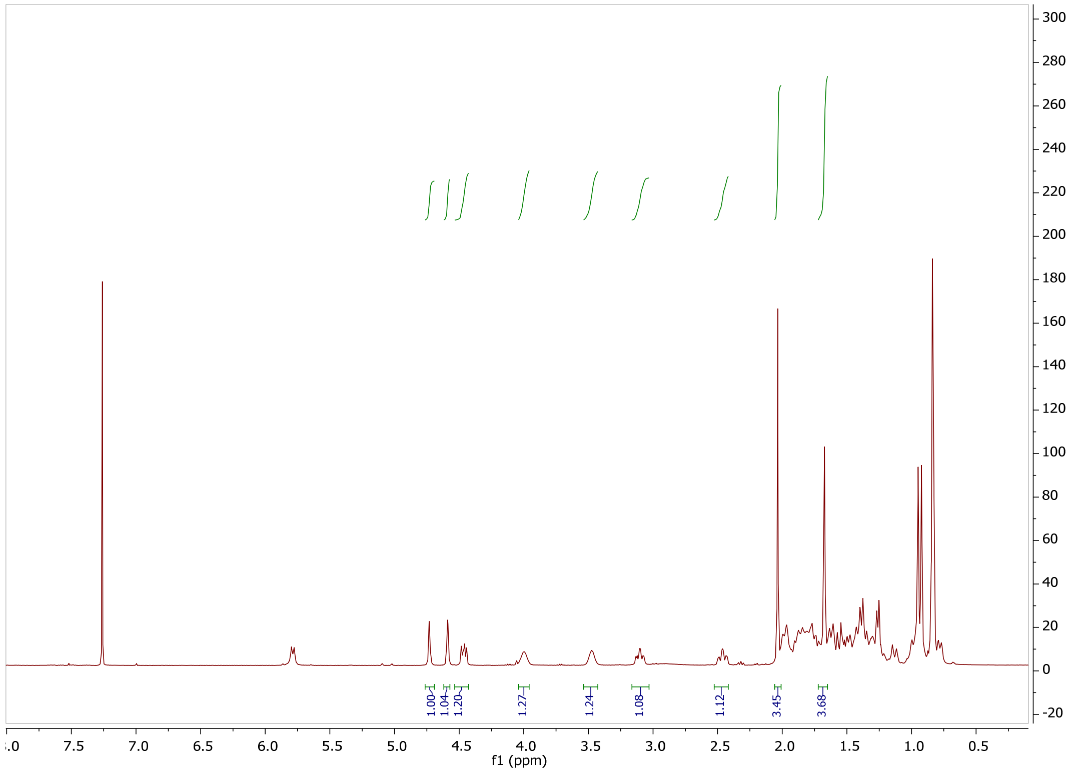

^{13}C APT-NMR (101 MHz, $CDCl_3$)

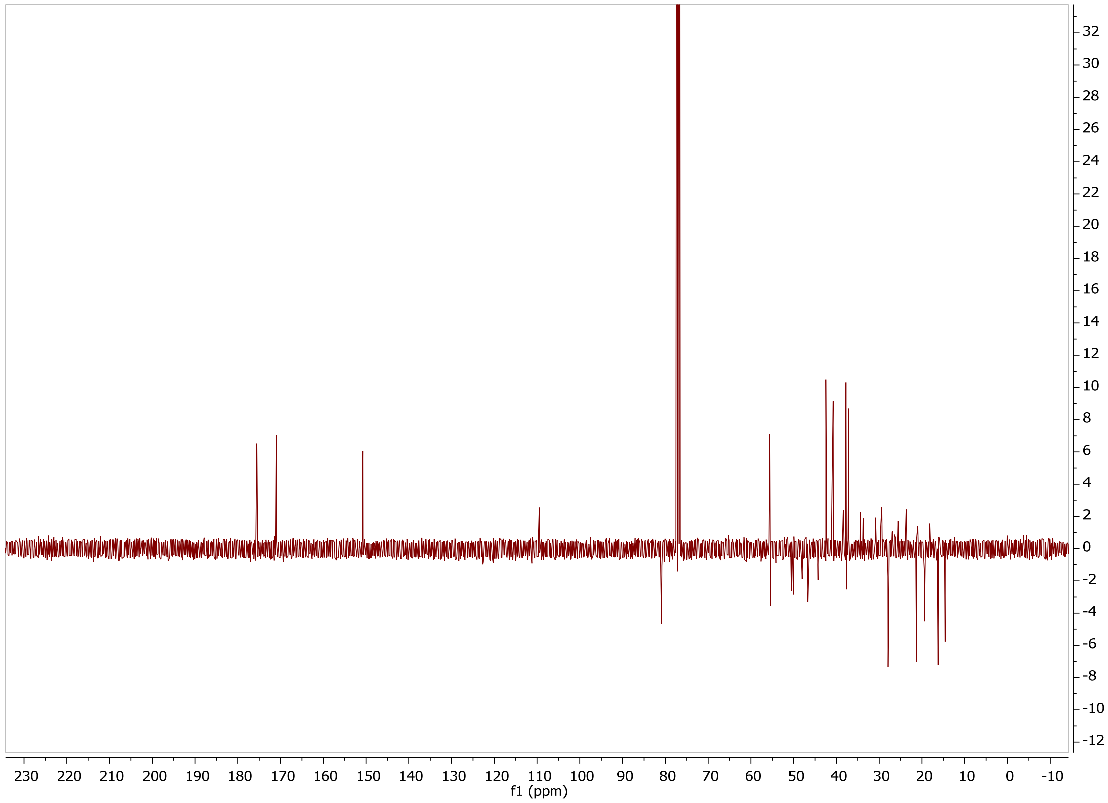

Spectra of 15

^{1}H NMR (400 MHz, $CDCl_3$)

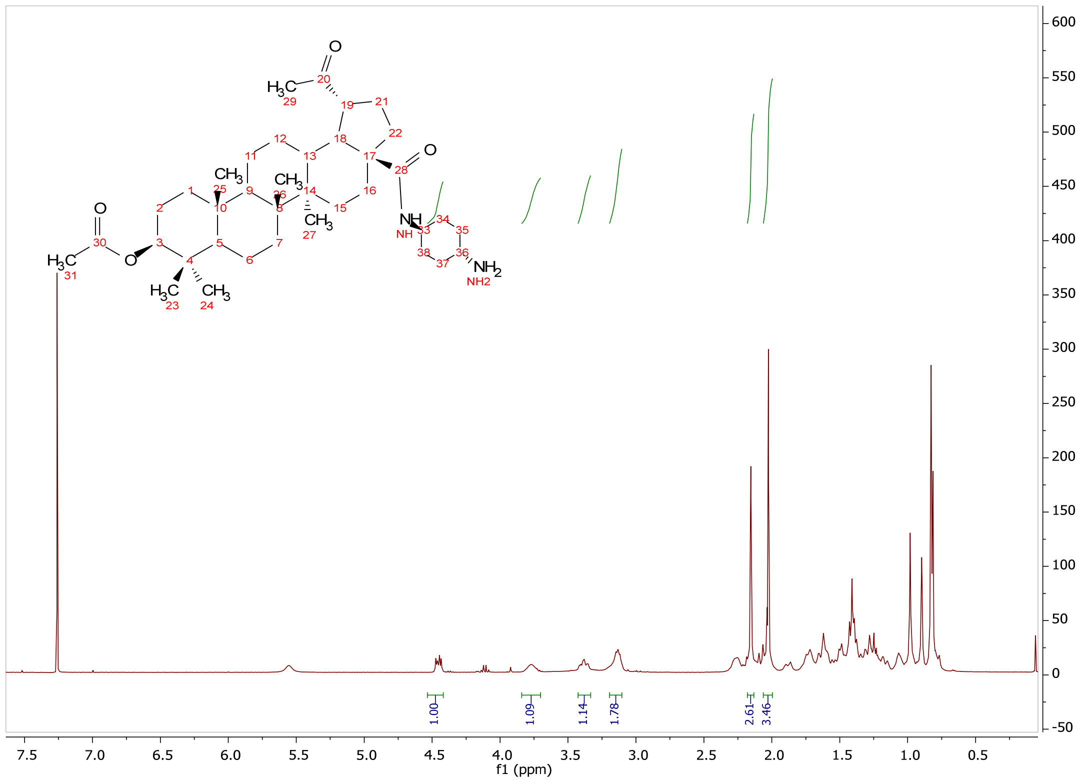

^{13}C APT-NMR (101 MHz, $CDCl_3$)

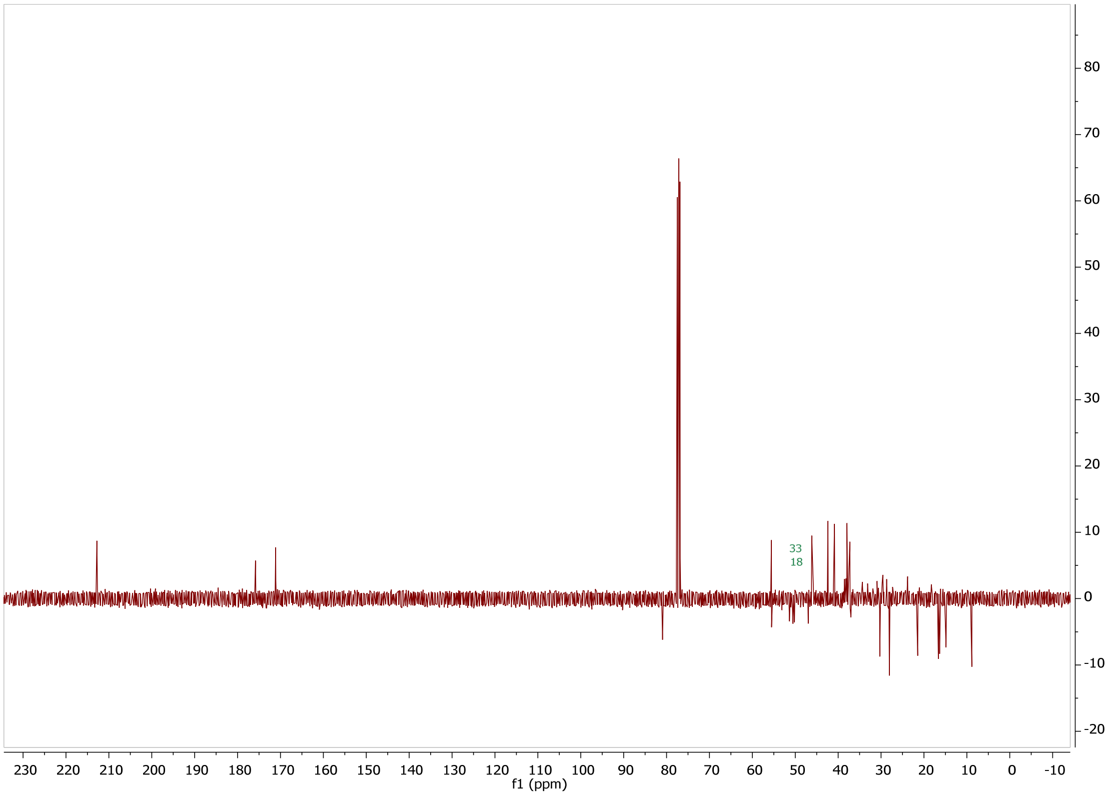

Spectra of 16

^{1}H NMR (500 MHz, $CDCl_3$)

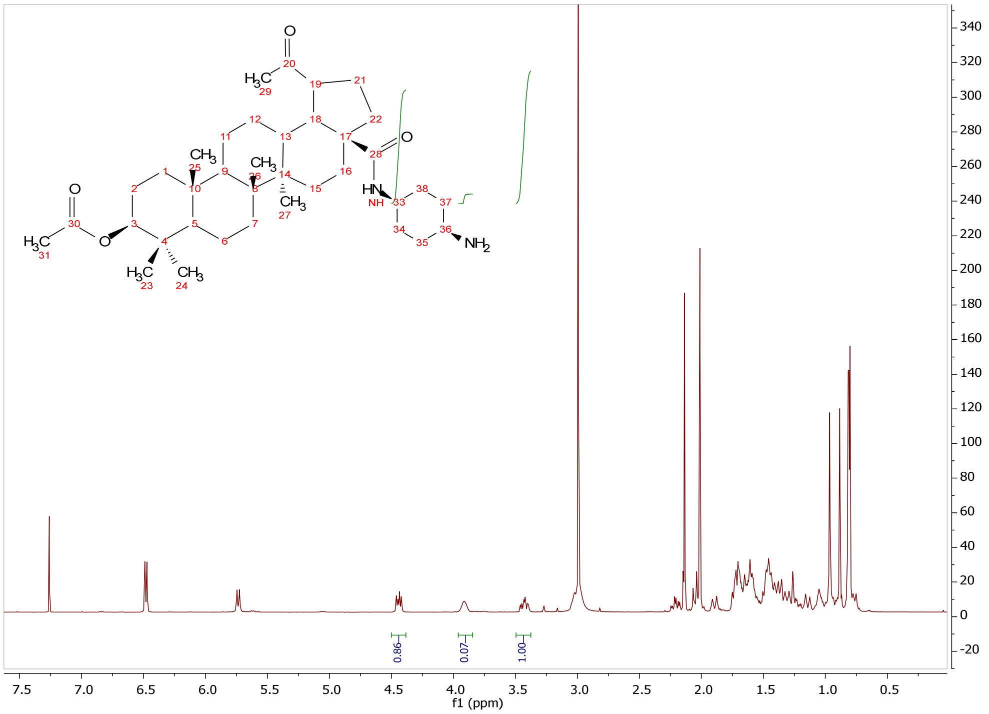

^{13}C APT-NMR (126 MHz, $CDCl_3$)

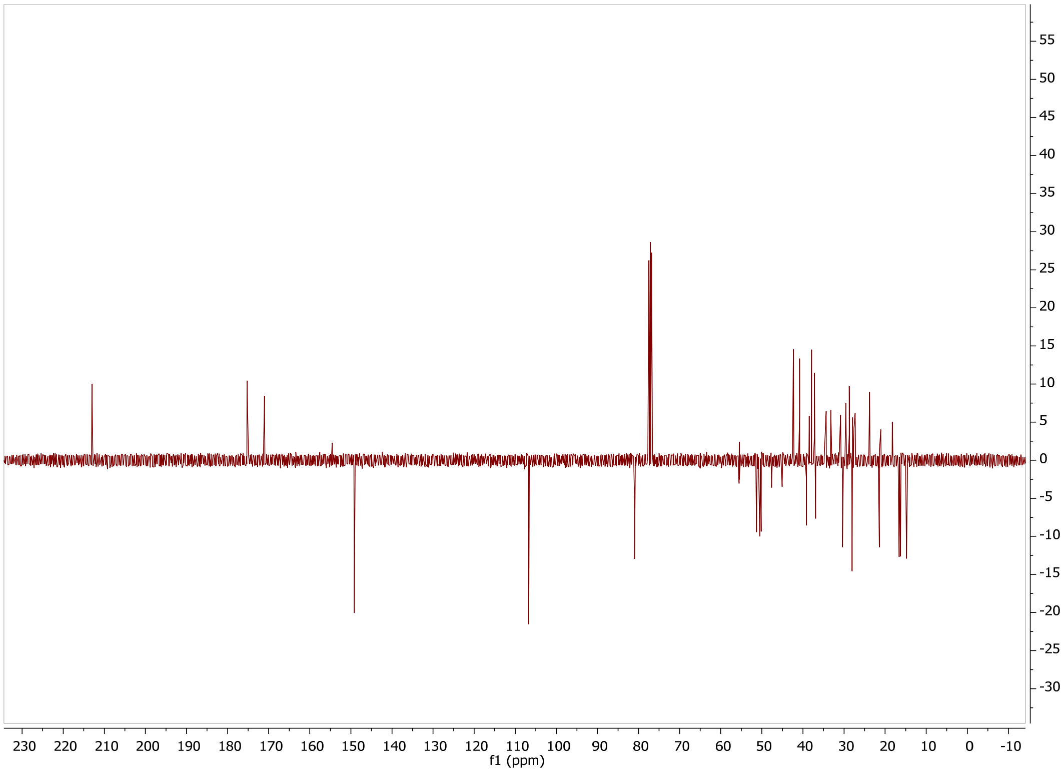

(3β)-28-(1,4-Diazabicyclo[3.2.2]nonyl-4-yl)-28-oxoolean-12-en-3-yl acetate (25)

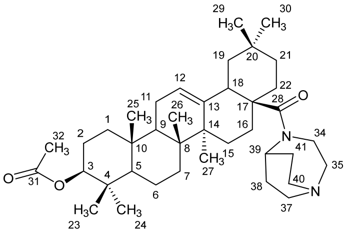

^{1}H NMR (400 MHz, $CDCl_3$):

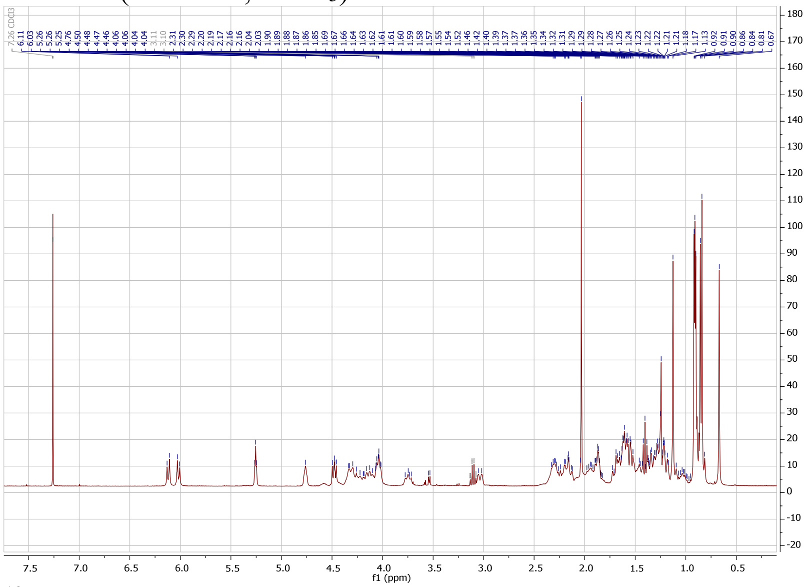

^{13}C APT-NMR (101 MHz, $CDCl_3$):

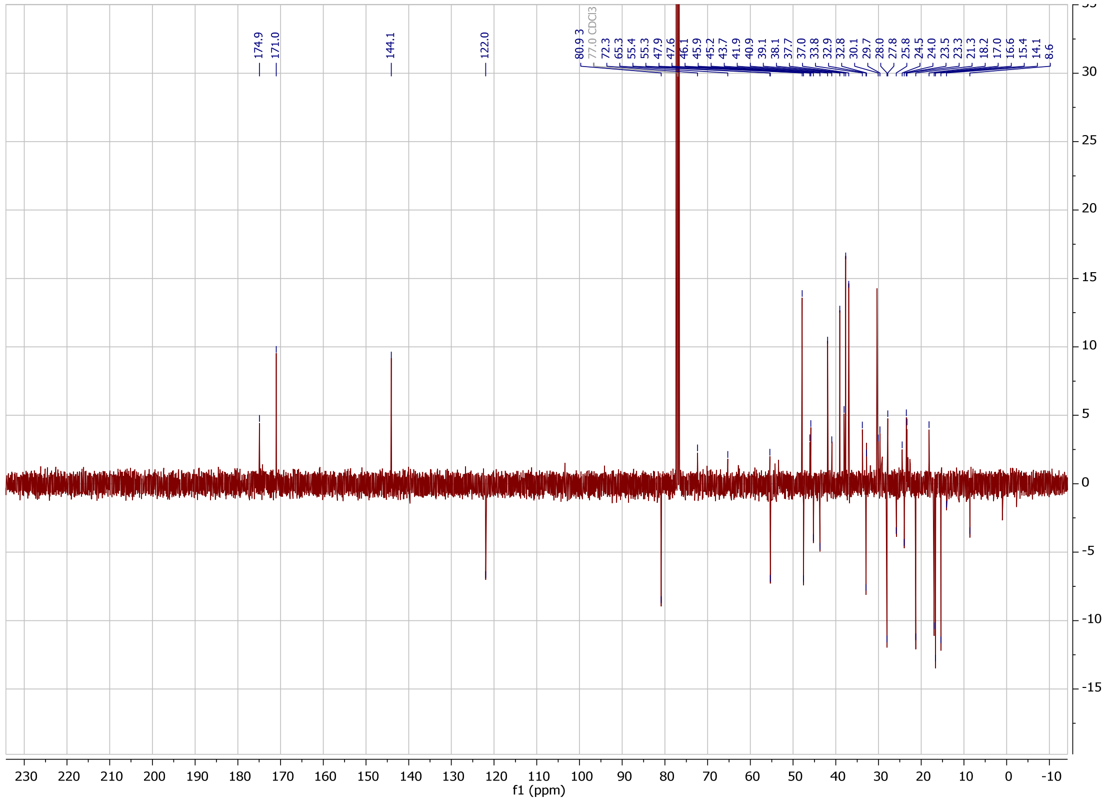

(3β)-28-(1,4-Diazabicyclo[3.2.2]non-4-yl)-20,28-dioxo-30-norlupan-3-yl acetate (27)

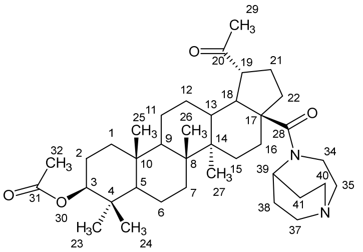

^{1}H NMR (500 MHz, $CDCl_3$):

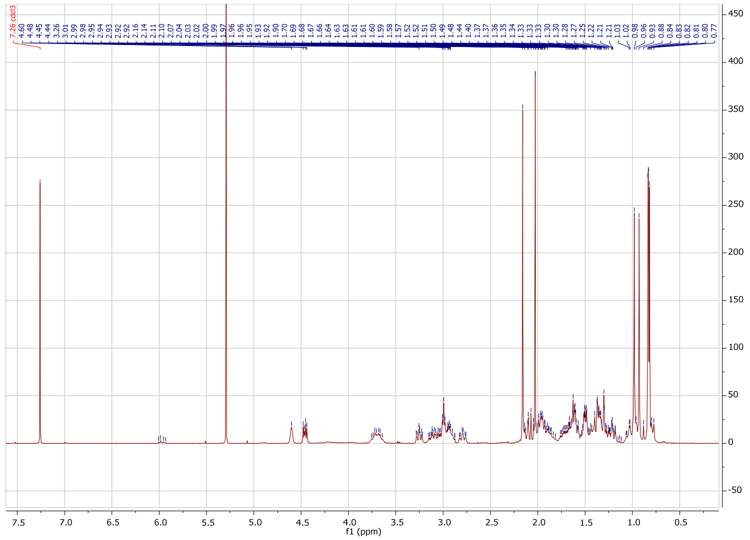

^{13}C APT-NMR (126 MHz, $CDCl_3$):

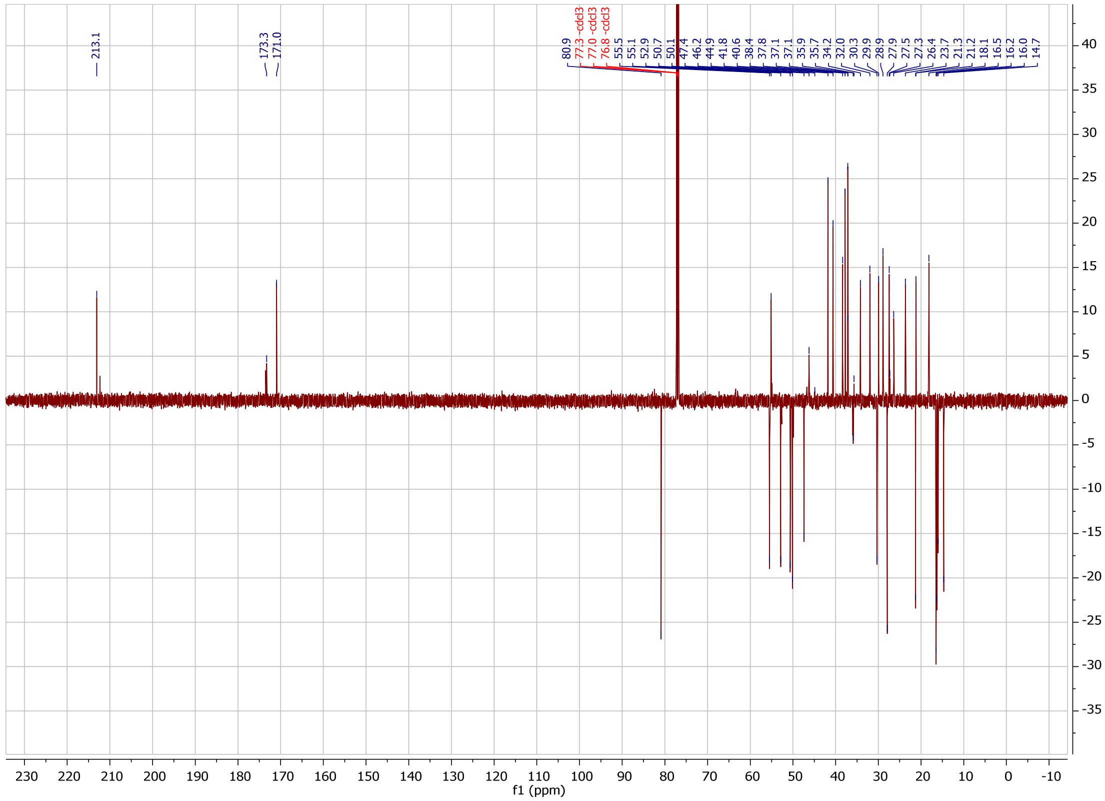

(3β)-28-(1,3-Diazabicyclo[3.2.2]nonyl-3-yl)-28-oxoolean-12-en-3-yl acetate (28)

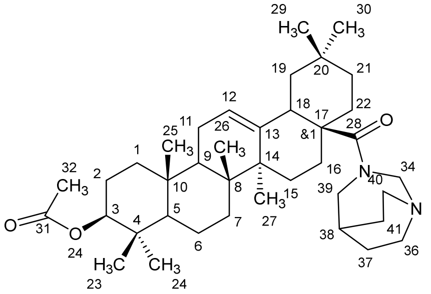

^{1}H NMR (400 MHz, MeOH-d4):

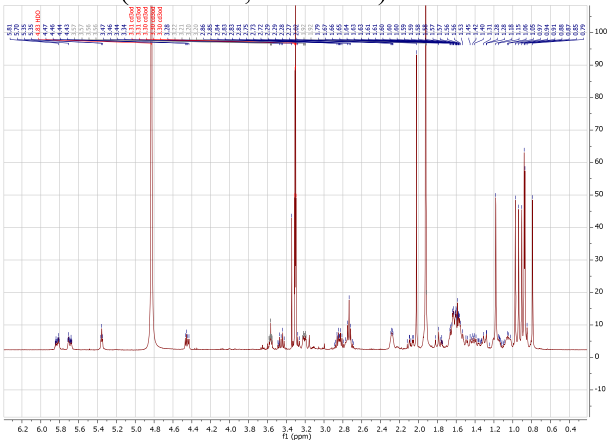

^{13}C APT-NMR (101 MHz, MeOH-d4):

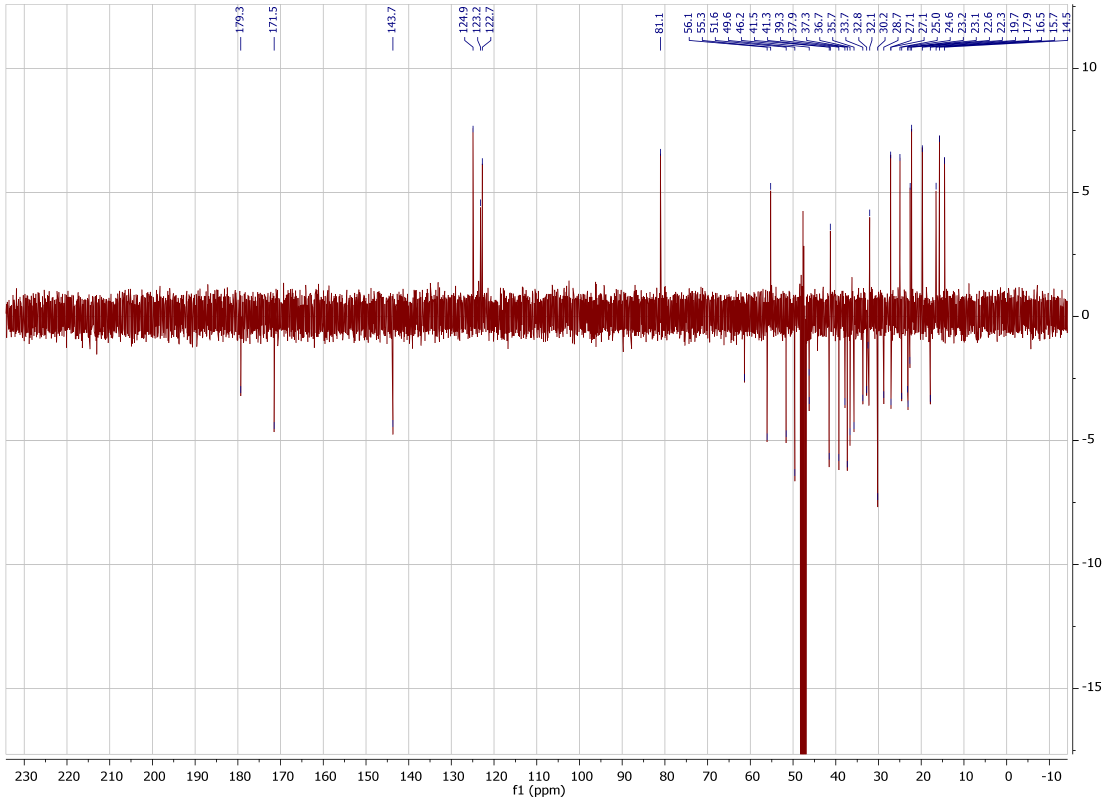

(3β)-28-(1,3-Diazabicyclo[3.2.2]nonyl)-3-yl)-28-oxours-12-en-3-yl acetate (29)

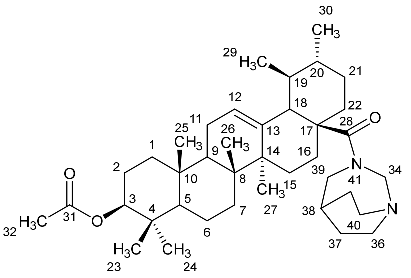

^{1}H NMR (500 MHz, $CDCl_3$):

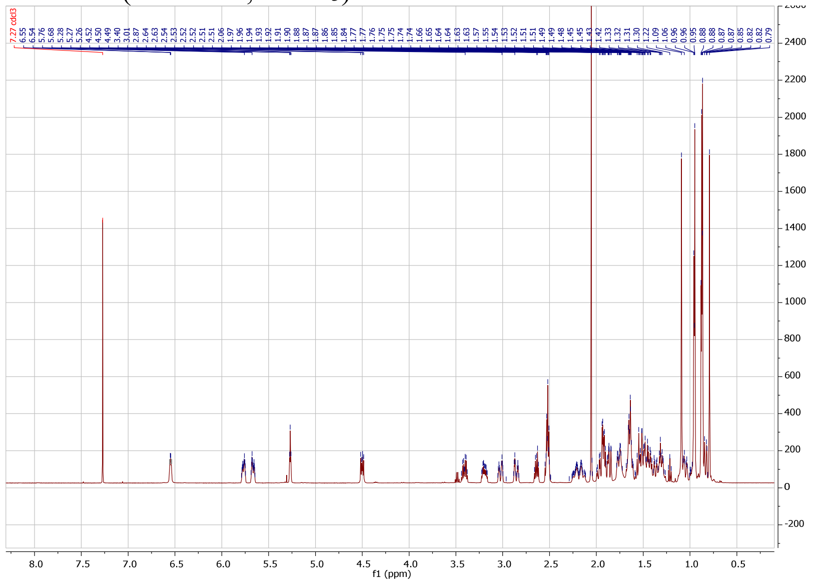

^{13}C APT-NMR (126 MHz, $CDCl_3$):

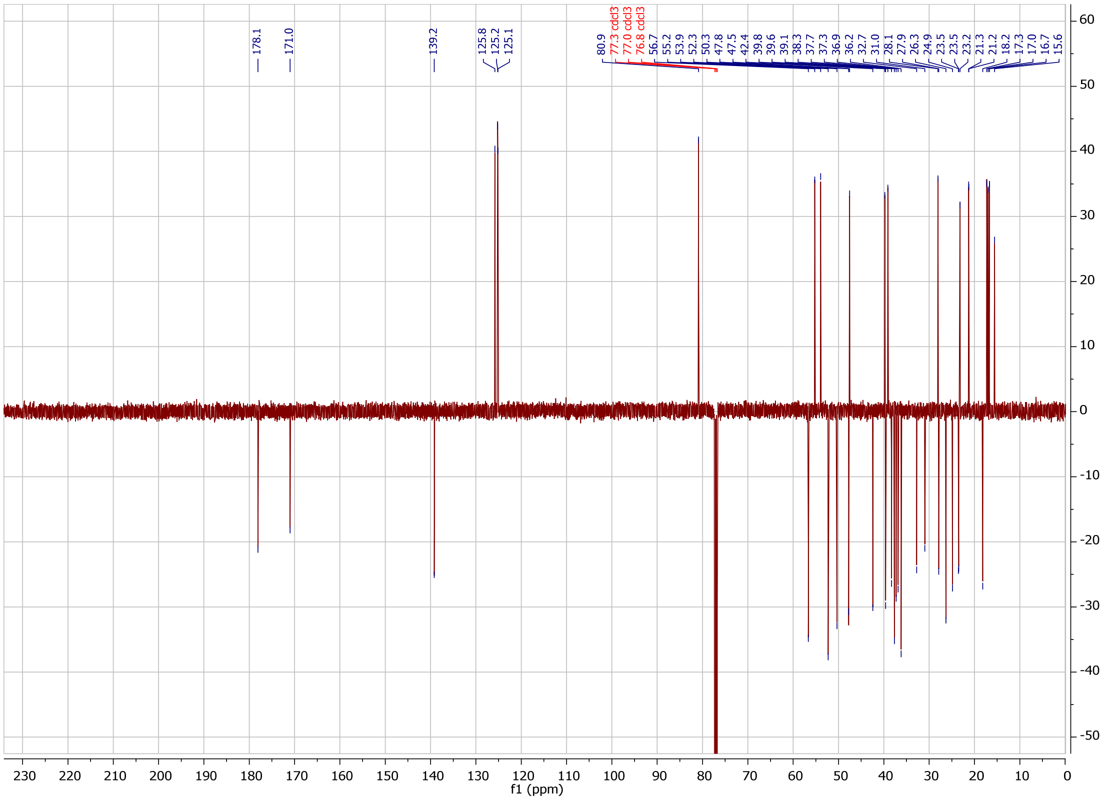

(3β)-28-(1,3-Diazabicyclo[3.2.2]non-3-yl)-28-oxolup-20(29)-en-3-yl acetate (30)

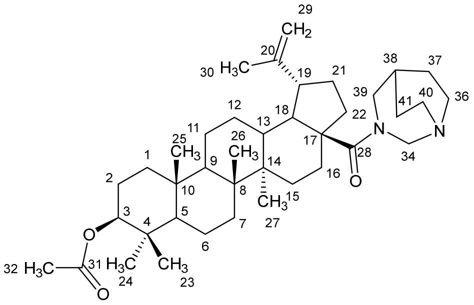

^{1}H NMR (400 MHz, $CDCl_3$):

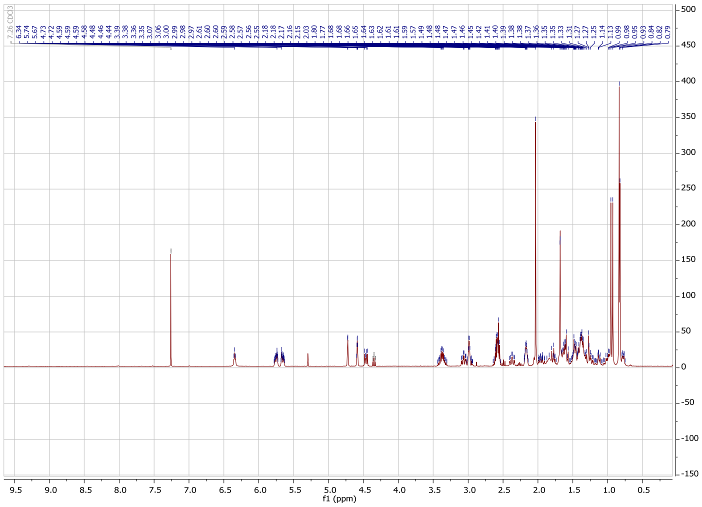

^{13}C APT-NMR (126 MHz, $CDCl_3$):

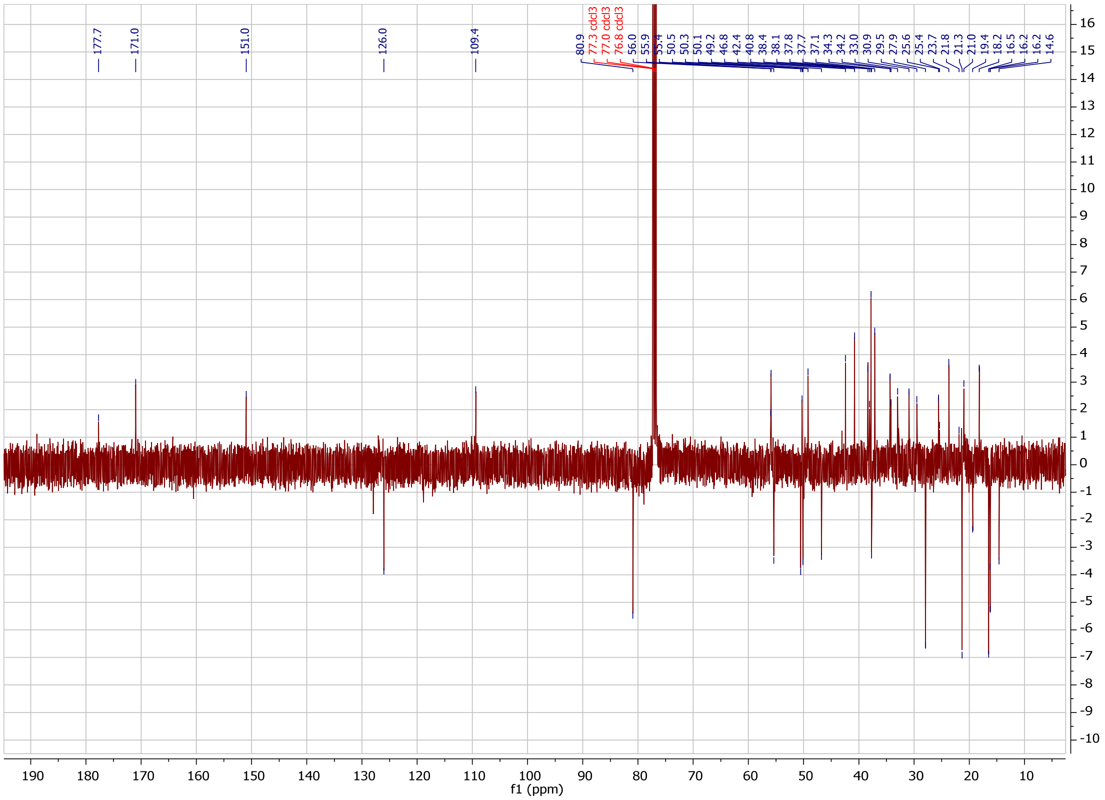

(3β)-28-(1,3-Diazabicyclo[3.2.2]non-4-yl)-20,28-dioxo-30-norlupan-3-yl-acetate (31)

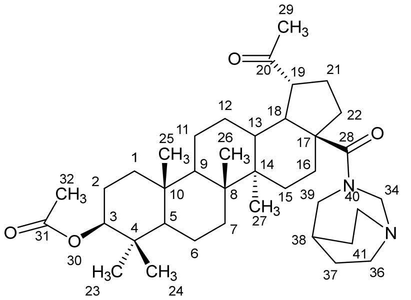

^{1}H NMR (500 MHz, $CDCl_3$):

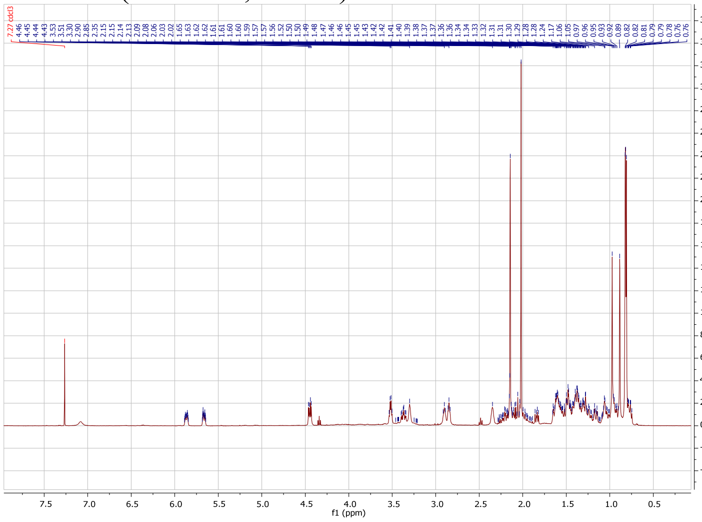

^{13}C APT-NMR (126 MHz, $CDCl_3$):

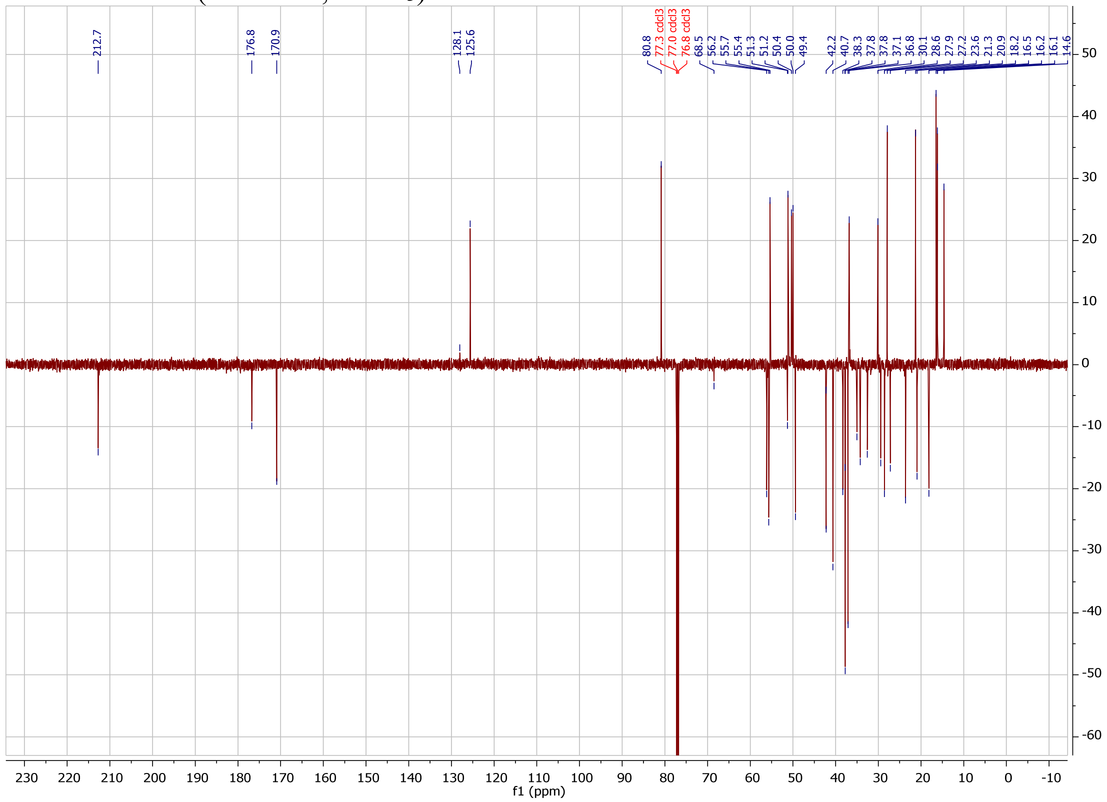

Selected IR (ATR) Spectra

ATR-IR spectrum of **9**

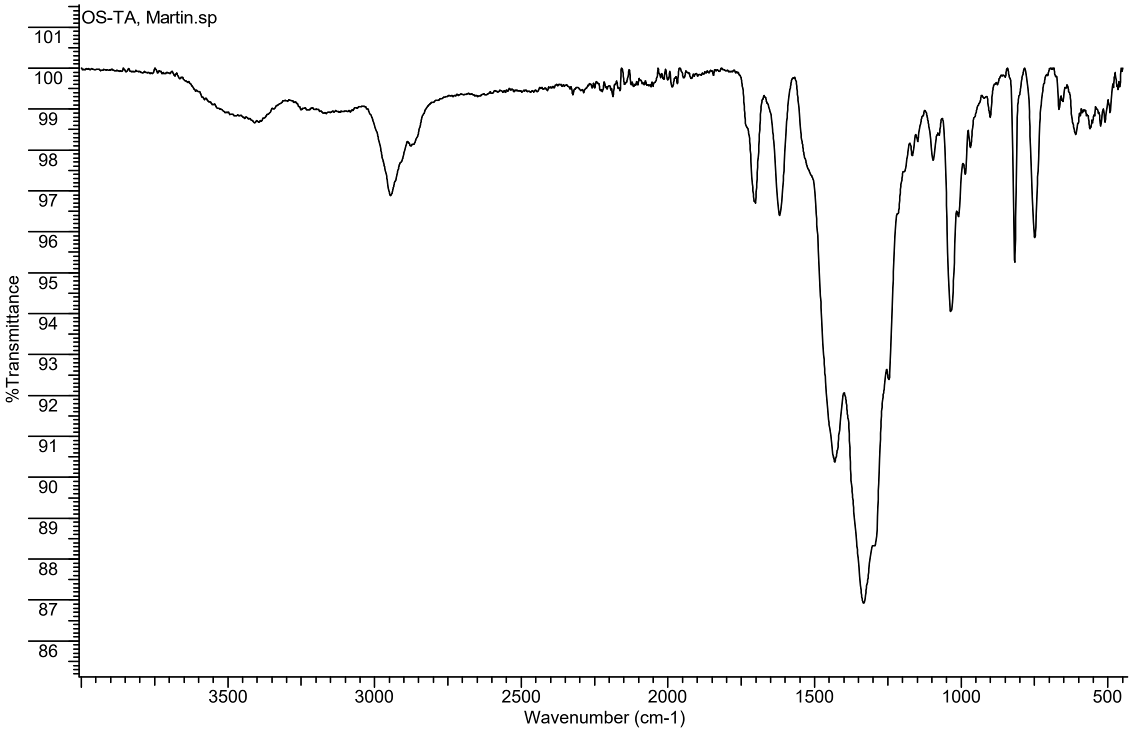

ATR-IR spectrum of **10**

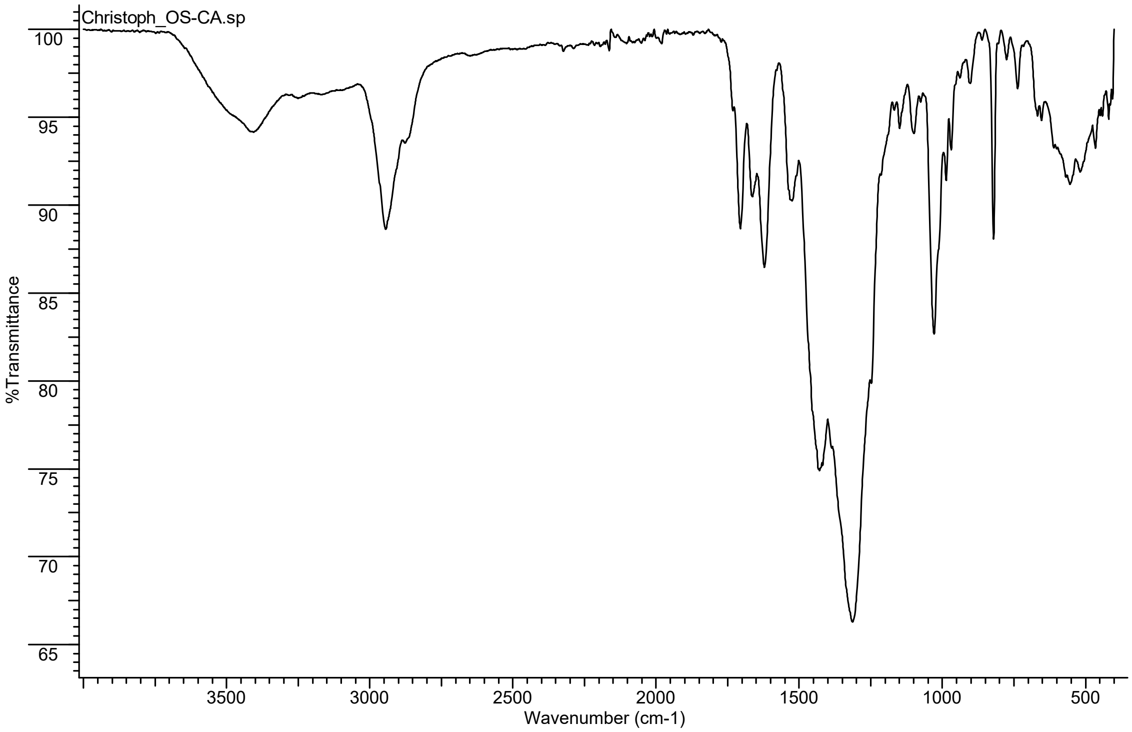

ATR-IR spectrum of **11**

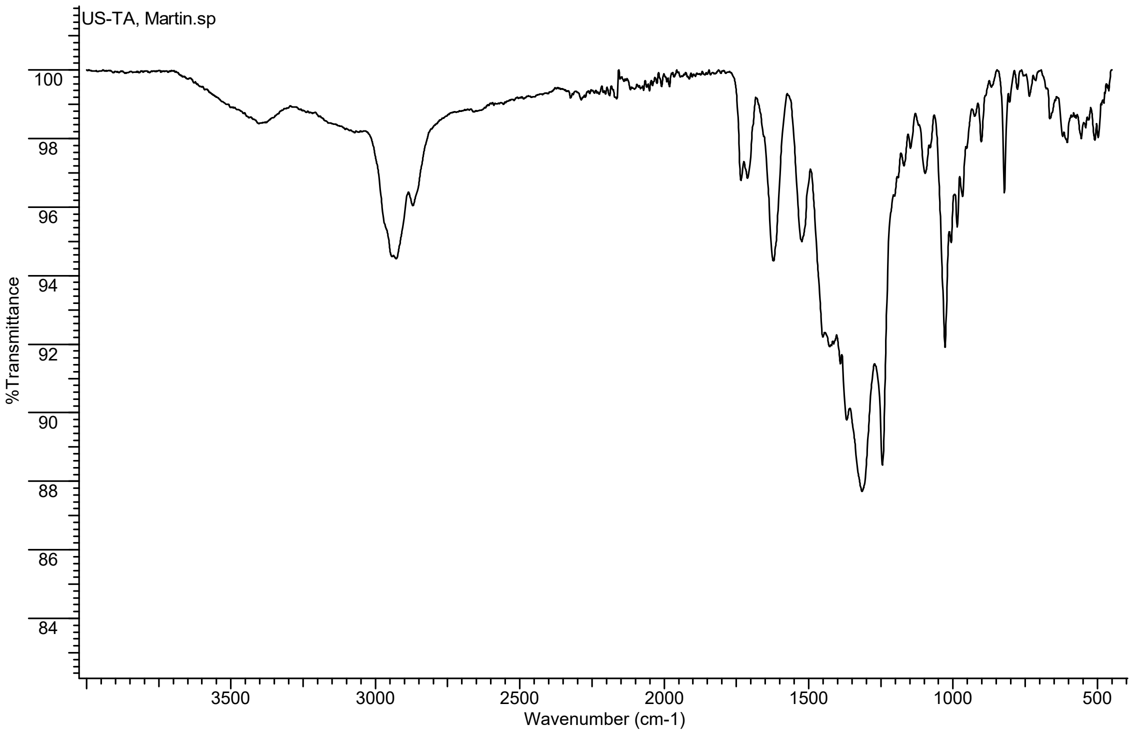

ATR-IR spectrum of **12**

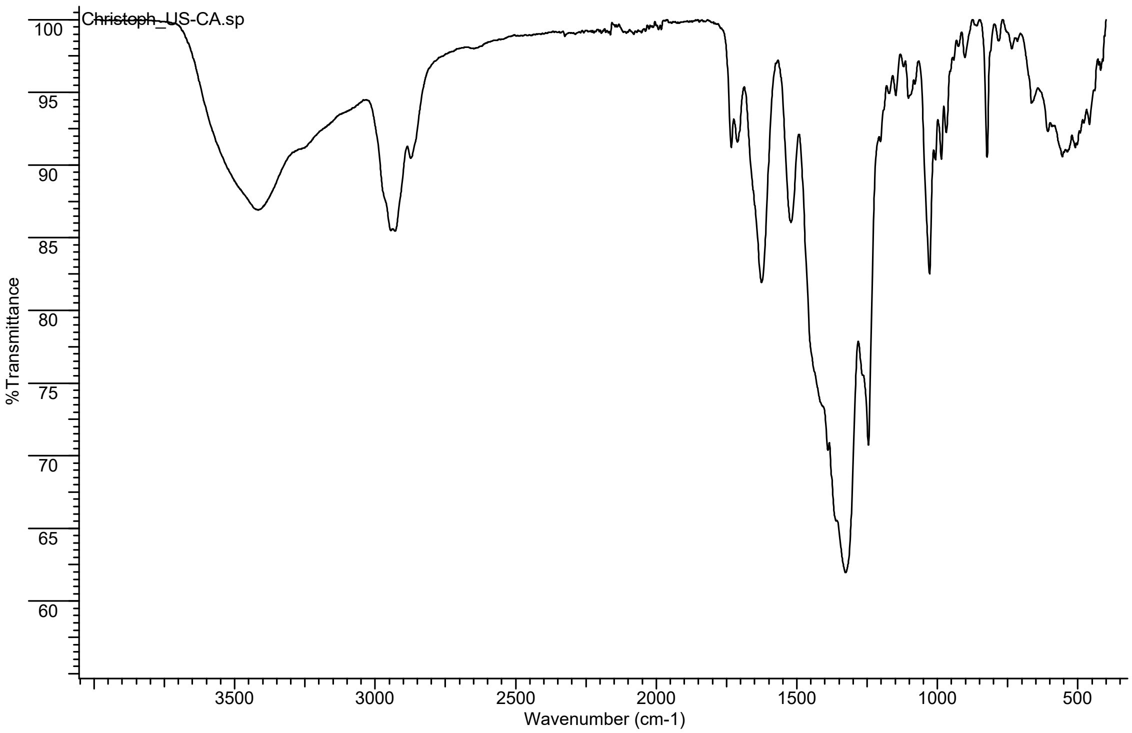

ATR-IR spectrum of **13**

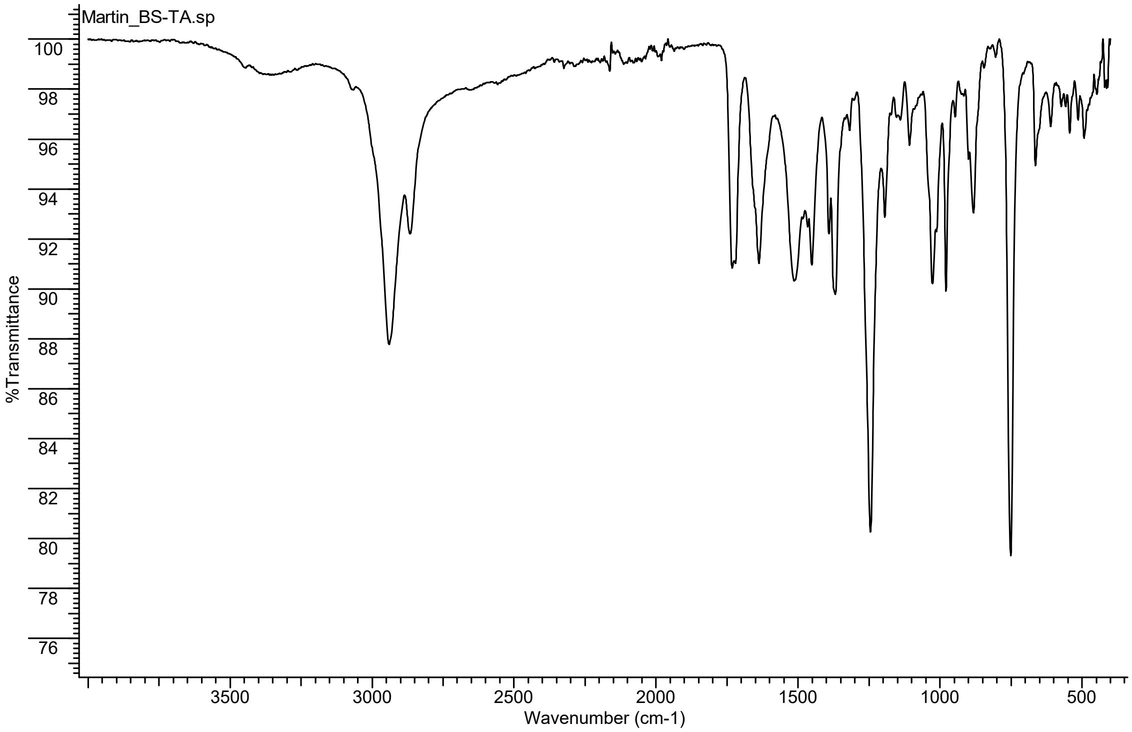

ATR-IR spectrum of **14**

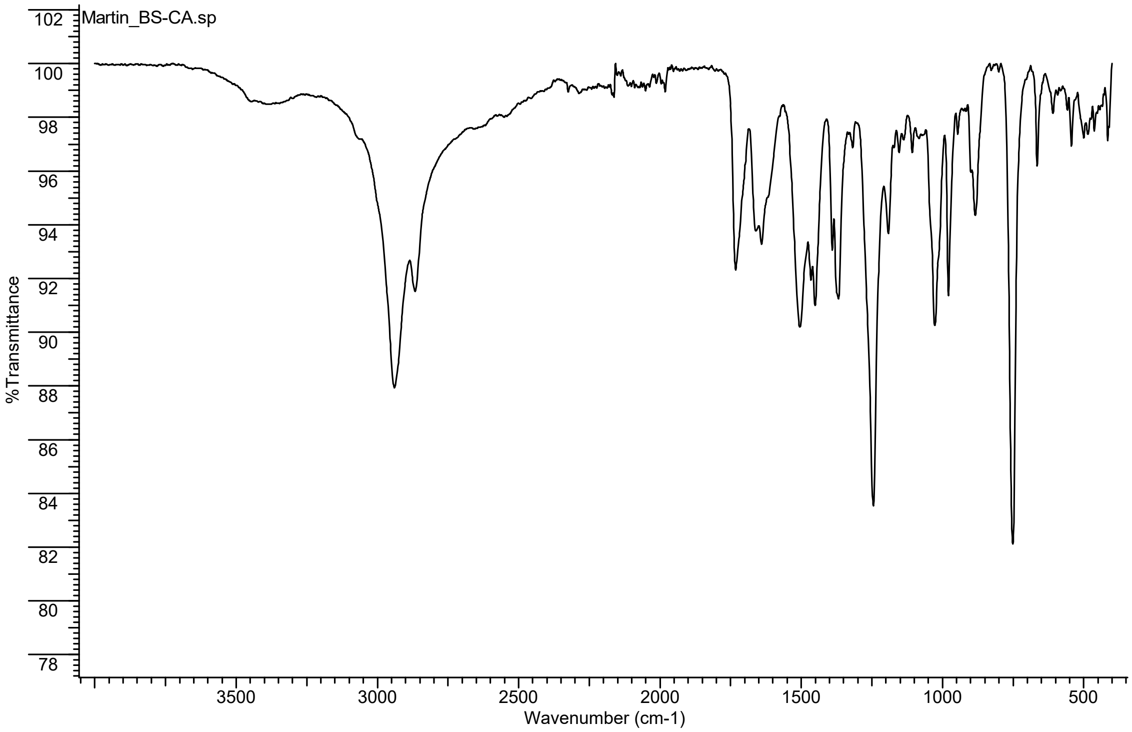

ATR-IR spectrum of **15**

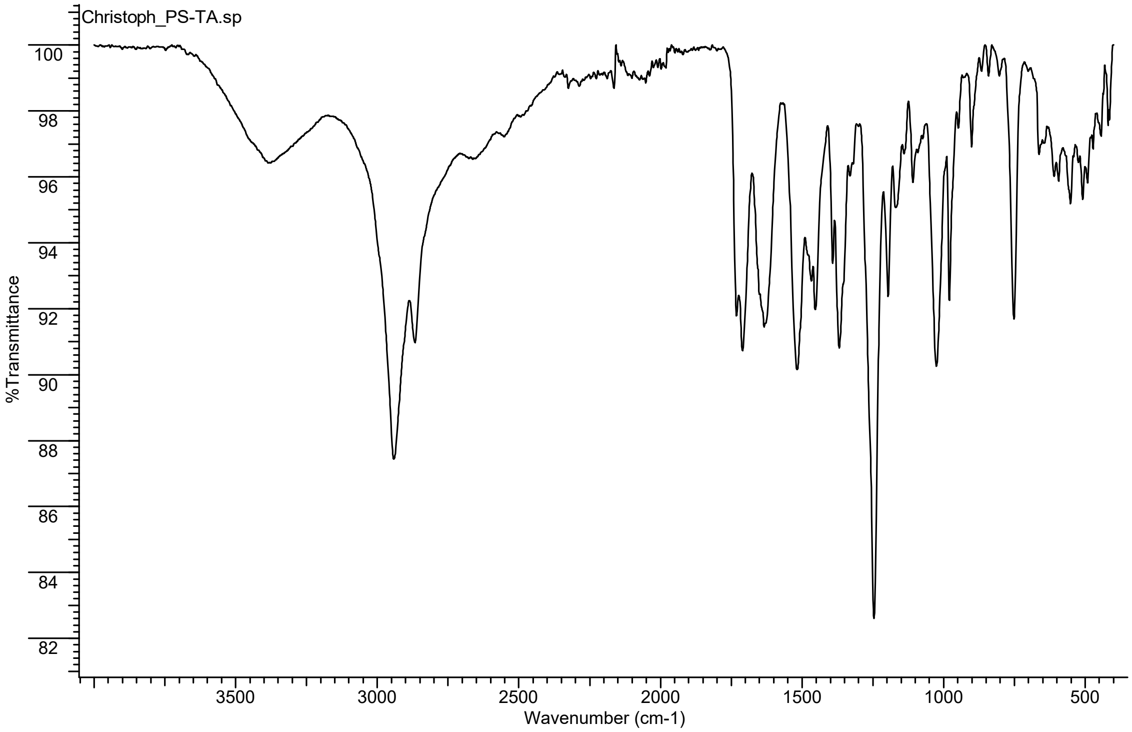

ATR-IR spectrum of **16**

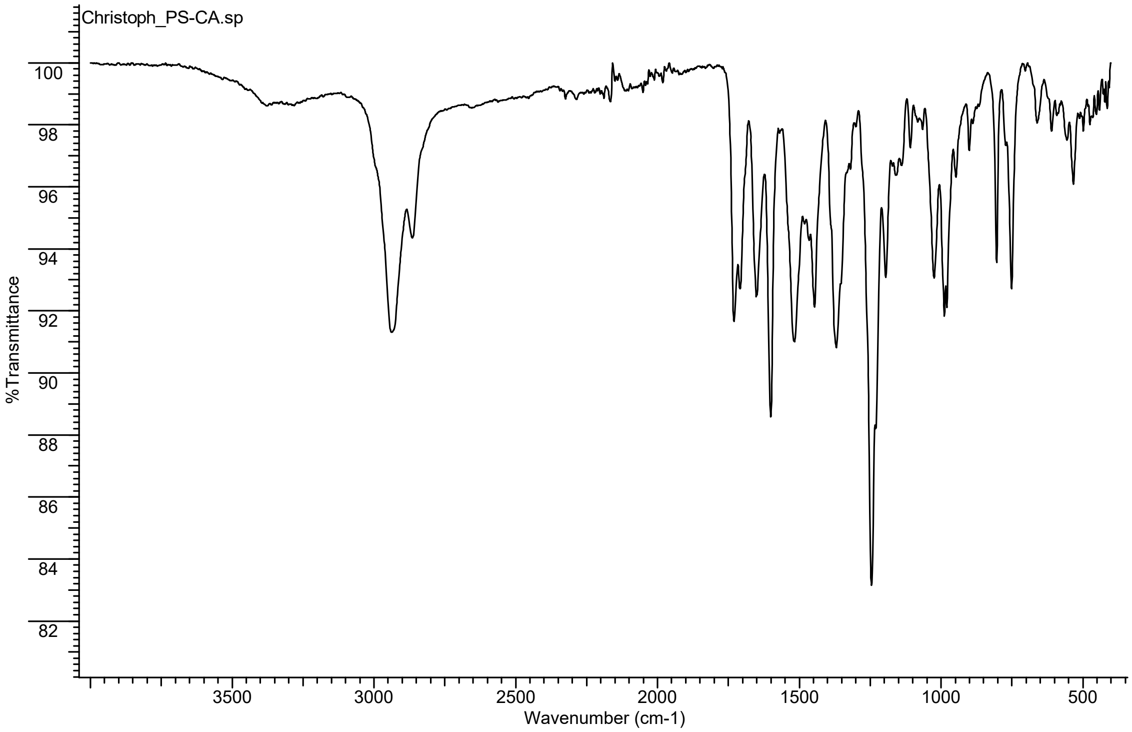

Supplement: Supplementary file 1 [file molecules-26-02102-s001.pdf]
